# Spatial Modeling of Vesicle Transport and the Cytoskeleton: The Challenge of Hitting the Right Road

**DOI:** 10.1371/journal.pone.0029645

**Published:** 2012-01-12

**Authors:** Michael Klann, Heinz Koeppl, Matthias Reuss

**Affiliations:** 1 Automatic Control Laboratory, Swiss Federal Institute of Technology Zurich, Zurich, Switzerland; 2 Center Systems Biology, University of Stuttgart, Stuttgart, Germany; Institut Curie, France

## Abstract

The membrane trafficking machinery provides a transport and sorting system for many cellular proteins. We propose a mechanistic agent-based computer simulation to integrate and test the hypothesis of vesicle transport embedded into a detailed model cell. The method tracks both the number and location of the vesicles. Thus both the stochastic properties due to the low numbers and the spatial aspects are preserved. The underlying molecular interactions that control the vesicle actions are included in a multi-scale manner based on the model of Heinrich and Rapoport (2005). By adding motor proteins we can improve the recycling process of SNAREs and model cell polarization. Our model also predicts that coat molecules should have a high turnover at the compartment membranes, while the turnover of motor proteins has to be slow. The modular structure of the underlying model keeps it tractable despite the overall complexity of the vesicle system. We apply our model to receptor-mediated endocytosis and show how a polarized cytoskeleton structure leads to polarized distributions in the plasma membrane both of SNAREs and the Ste2p receptor in yeast. In addition, we can couple signal transduction and membrane trafficking steps in one simulation, which enables analyzing the effect of receptor-mediated endocytosis on signaling.

## Introduction

The organization of metabolic reactions and protein synthesis in eukaryotic cells requires complex machinery that maintains the creation and functionality of specialized compartments and controls the specific subcellular location of the respective proteins [1], [2]. The different membrane enclosed compartments (Endoplasmic Reticulum (ER), Golgi stacks, or Endosomes) form a dynamically linked network in which vesicles deliver cargo molecules from donor to target compartments [3]–[5].

**Figure 1:**
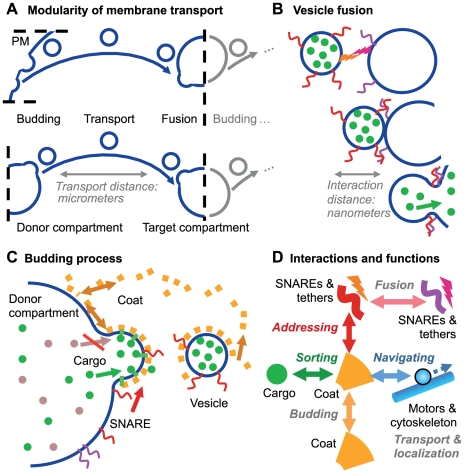
Modularity and Interactions of Vesicle Transport. A: Each transport step between two compartments (or the plasma membrane) can be seen as an individual module. Each module contains the budding, transport, and fusion step. B: Vesicle fusion is mediated by tethering factors and SNAREs. These molecules can only interact if the vesicle is in close vicinity of the target compartment. C: The budding process involves the formation of a coat (cf. [46]) and the loading of the desired cargo and SNARE molecules into the vesicle. D: Interactions between the molecules of the vesicle machinery. Each class of molecules/interactions can also be linked to a distinct function (see also Table S1). For each interaction a set of kinetic parameters has to be assigned. The total set of interactions between different subtypes of *‘coats’, ‘snare’, ‘cargo’*, and *‘motors’* can be broken into the subset of subspecies and interactions governing a given membrane trafficking connection between two compartments. In the principle of the Heinrich and Rapoport [12] model *‘coat A’* binds to *‘compartment 1’*, selects *‘cargo 1’* and *‘snare X’* into a vesicle which fuses via the strong *‘snare X-Y’* interaction to *Ôcompartment2’*. A second module, responsible for the reverse transport, is respectively set on the strong *‘compartment 2’-‘coat B’-cargo 2′-snare V′-‘snare U-V’* interaction. The directed transport with motor proteins requires adding *‘motor 1’* going from *‘compartment 1’* towards *‘compartment 2’* and the reverse *‘motor 2’* accordingly. These *‘motors’* represent for instance Kinesin and Dynein that walk along microtubules in different directions.

The key features of vesicle transport are the accurate selection of only the desired molecules into the vesicles and the transport of the vesicle towards the correct target through the crowded intracellular environment [6]. While sorting depends on specific (short range) molecular interactions between the proteins forming a vesicle [3], [7], the navigation through the cell requires a long-range orientation (cf. Figure 1 a–c) [8]. Motor proteins can pull vesicle along cytoskeleton tracks [9], [10]. This allows the directed motion towards the target, given that the vesicle happens to run on the right track. Considering the large number of cytoskeleton filaments and furthermore their dynamics, finding the right way through the cell is not a trivial task [11]. But also the probability to hit a desired target only by diffusion is rather small. The present work investigated the principal interactions of the transport process and the connecting cytoskeleton structures which guarantee that a vesicle is not lost in space.

For a rigorous analysis, the large network can be broken into small units. Each vesicle transport step between two compartments forms such an elementary module as depicted in Figure 1a [12], [13]. One module includes vesicle budding at the donor compartment, transport, and the fusion process at the target compartment. During their lifetimes compartments and vesicles can maturate and develop into another compartment, for instance the early into the late endosome [14].

In principle, each vesicle and compartment is an autonomous entity. The initial state determines the temporal development of its location, internal biochemical conversions, and interactions with other objects in the cell. This especially holds for the key proteins of the vesicle-vesicle interaction, i.e. the fusion process. Vesicle fusion is initiated by a docking and tethering state induced by tethering factors [15]. Subsequently the binding of SNARE (Soluble NSF Attachment protein REceptors) proteins connects both membranes and promotes the eventual fusion via a cis-trans-conversion (cf. Figure 1 b). The SNARE proteins can be subdivided into the v-SNAREs in the vesicle membrane and the t-SNAREs at the target compartment [16].

Accordingly, the v-SNAREs have to be loaded into the vesicle during the budding process as shown in Figure 1 c [12]. The vesicle itself is created by the polymerization of a coat around it, which forms its shape and selects the cargo molecules via transmembrane domains. This coat consists of a variety of proteins, can be classified as COPI, COPII, or clathrin coat, and shows a modular design [17]–[19]. The variety of proteins involved in the coat formation and cargo selection on the one hand and the need to simplify this complexity in order to build a full-scale model of the vesicle machinery on the other hand can be accounted for by defining each coat complex as just one generic molecule. We define different coat molecules as subtypes of the coat molecule class, which can vary in their affinity for cargo, SNARE, etc. molecules (just like the real complexes vary in their protein composition). Thus the relevant aspect of the complexity of the coat is preserved in our model. The two-compartment model of Heinrich and Rapoport (2005) likewise uses coat A and B and their different preferences for different compartments, cargo and SNAREs.

This ODE-model of Heinrich and Rapoport [12] was already able to generate nonidentical compartments and to facilitate the sorting of molecules. However, it omits the spatial aspects of vesicle transport, tracking only number, size, and state of the compartments - like the models of Gong et al. [20] and Brusch and Deutsch [21]. The most recent model of Birbaumer and Schweitzer [22] covers the spatial aspects with an agent-based simulation, but replaces the cytoskeleton by a potential/force field directing the vesicles and neglecting the molecular details of the budding and fusion machinery. Other models include the spatial aspect using a continuous flow approach to describe the vesicle flux, thus neglecting the discrete properties of individual vesicles [23] or only cover sub-problems like budding [24], [25] and fusion [26].

Especially when spatial models are considered, the interactions with motor proteins and cytoskeleton filaments have to be considered for navigating the vesicles through the cellular space. Accordingly we propose to include the affinity of motor proteins to the coats so that they are added to the vesicles during the budding process [23]. Figure 1 d shows the complete network of interactions between the molecule species that are involved in membrane trafficking.

The aim of the present work is to integrate and condense the present knowledge into a 4D spatio-temporal agent-based model. The virtual three-dimensional cell which is set up in order to model vesicle transport contains cytoskeleton structures and all necessary molecule species to drive the membrane trafficking machinery. The structured and event based approach also preserves the inherent stochasticity equal to the stochastic noise and fluctuations in the real number of vesicles. The limitation of agent/molecular interactions to relevant interactions of the model and the separation into interactions within vesicles and between vesicles in a multi-scale manner still keeps the simulation tractable despite the overall complexity. The introduced modularity of the vesicle transport network further improves the handling and allows an easy scale-up from a simple two-compartment setup towards a model containing all compartments.

## Results and Discussion

### Transport and navigation

The challenge in the discrete and spatially segregated model arises from the task to guide vesicles towards distant targets - based on local molecular interactions. We acknowledge that by diffusion each vesicle can explore the whole cell and thus eventually will find its target. But that would also mean that all vesicles (presumably with precious cargo) are uniformly distributed in the cell and not just present at the target area. In addition, microscopy time series images indicate, that vesicles move directly to their target [10]. The observed inhomogeneous vesicle and compartment distributions in the cell require that they are guided and sorted towards the different locations.

In the following, the transport properties of a simple two-compartment system have been evaluated within several virtual cytoskeleton network architectures as well as with and without motor proteins. The two initial compartments are sufficiently loaded with cargo, SNAREs, and motor proteins so that functional vesicles can be formed during the budding process in the simulation (see Methods for description of the simulation). In order to investigate the influence of the diffusion coefficient, it is set to (i) 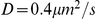 and (ii) 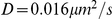 for vesicles with a diameter of 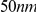. The slower diffusion coefficient could for instance arise from transient binding effects. All other parameters are given in Text S2.

**Figure 2:**
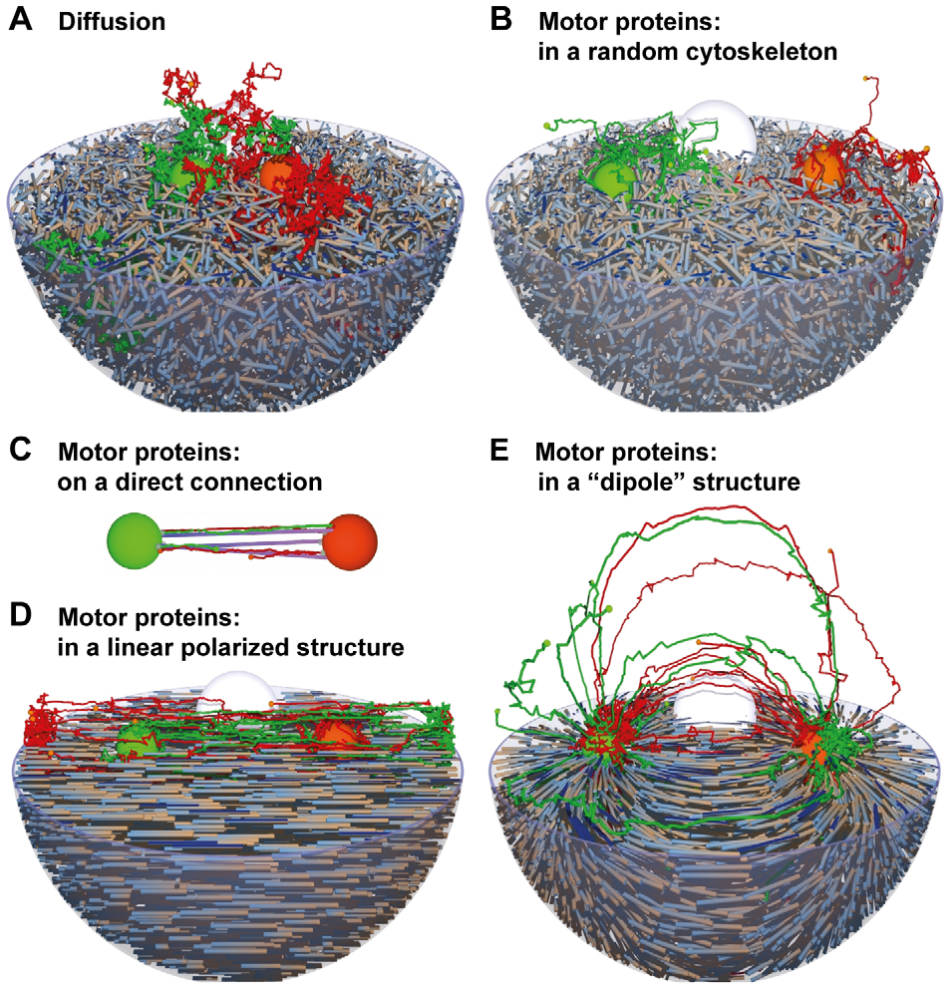
Spatial Aspects of Vesicle Transport in a Two Compartment System. Spatial aspects of vesicle transport in a two compartment system: Comparison of diffusion and transport with motor proteins in different cytoskeleton structures. Vesicles and their paths are shown in similar colours as the donor compartment. Orange vesicles bud from the orange compartment 1, targeted for the green compartment 2. Green vesicles go into the opposite direction. Parameters are given in Text S2.

Figure 2 shows sample vesicle paths in these systems:

- Diffusion alone distributes the vesicles everywhere in the cell. Only a few vesicles reach the correct target compartment (by chance) within a reasonable time - despite the fact, that the compartments are closer together than in all other setups (in agreement with the findings of [27]. For the slowly diffusing set of vesicles even after 600 seconds of the simulation, in which constantly new vesicles have been formed, only 76 fusion events have been recorded, of which 70 were backward fusion events. The faster vesicles lead to better forward fusion characteristics because they leave their donor compartment faster.
- If the cytoskeleton is randomly arranged, vesicles following these tracks with motor proteins are not able to reach the target compartment either. The motion is still random, just on another scale. If the random network structure contains ‘sinks’, the vesicles will eventually end up there.
- In contrast, a direct connection leads to an optimal transport of the vesicles - they all reach their target directly (out of the first 400 fusion events only 39 backward fusions have been recorded, the average travel time was approx. 15 s and thus the fastest of all setups). It is worth noting, that the budding site has to be connected directly to the cytoskeleton filaments in this case. If the vesicle buds somewhere else, diffusion might drive it away from the track. Accordingly, the proteins anchoring the compartment to the cytoskeleton should have a connection to the vesicle budding machinery e.g. the coat molecules in this case. This is well in agreement with the findings of Kirk and Ward [28], reporting that there are special exit sites in the ER (for the ER to Golgi transport) and that they co-localize with microtubules. This isolated setup is functional, but the question remains, how this structure can be embedded into the cell.
- Interestingly, a linearly polarized cytoskeleton between the compartments does not lead to a high rate of fusion events. While the vesicles go into the right direction (but randomly switch the tracks), they often miss the target compartment and finally accumulate at the plasma membrane. After 600 seconds 130 fusion events have been recorded, after 900 seconds 217 events (130 forward, 87 backward).
- This leads to the conclusion, that a ‘good’ cytoskeleton structure focuses vesicles onto the target compartment. For a two-compartment system the respective structure could look like the electric field of two separated point charges with opposite charges at the position of the initial compartments. For this configuration nearly all vesicles reach their target compartment. After 616 seconds 400 fusion events have been recorded for the slower vesicle set, out of which only 127 fused with their donor compartment. Again the faster vesicle set has a better forward to backward fusion ratio because the escape process from the donor compartment is less diffusion limited.

**Figure 3:**
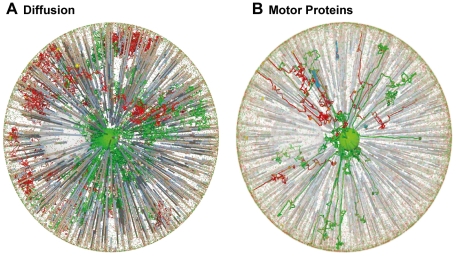
Spatial Aspects of Vesicle Transport in Endo- and Exocytosis. Spatial aspects of vesicle transport in endo- and exocytosis: Comparison of A diffusion and B transport with motor proteins (red: endocytic vesicles; green: recycling vesicles from the big green endosome in the centre).

Similar results are obtained in a two-compartment system consisting of the plasma membrane and an endosome in the centre of the cell. Endo- and exocytosis connect these compartments as shown in Figure 3. Again diffusion is not able to transport the vesicles towards the target. A radial cytoskeleton directly connects the endosome in the centre of the cell with the plasma membrane and thus provides a functional structure for vesicle transport with motor proteins. It is worth noting, that this ‘monopole’ structure corresponds to both, the ‘dipole’ structure (d) and the direct connection (c) of the previous two-compartment model due to the spherical symmetry. Obviously, if not just two large spheres but rather distributed compartments (like the distributed Golgi stacks) have to be connected, the structure has to be less focusing as well.

**Figure 4:**
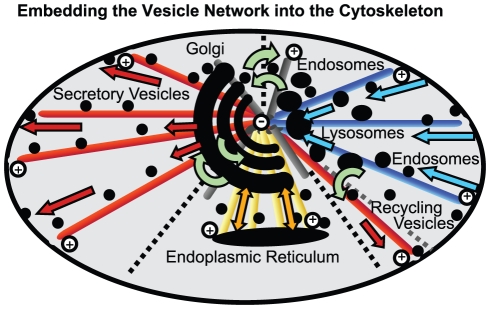
Embeding the Vesicle Transport Network in the Cytoskeleton. The connection from ER to Golgi, Golgi to plasma membrane and Plasma membrane to the lysosomes can be aligned with the radial structure of the microtubule network, which then has to be tri-partitioned. The challenge is the bridging of these partitions to fully connect the vesicle network. Note, that in this structure transcytosis requires changing the motor direction in the centre.

Either way of connecting compartments has its advantage: a direct connection is highly efficient. But if a vesicle happens to dissociate from it, the vesicle will diffuse away and will be lost in space. A cytoskeleton structure capturing and focusing diffusing vesicles is less specialized yet more robust. It can capture diffusing vesicles everywhere in the cell (or at least in a region of the cell) and guide them to the target. However for more than two compartments (and the cell has more than two), several fields are necessary (one for each direction). Then there will always exist a boundary between two possible directions of the global field. At this divide a further regulation needs to be introduced in order to control that vesicles end up on the correct side (cf. Figure 4).

Accordingly a functioning model cell requires the true to original reconstruction of the underlying microtubule connection between all compartments in order to provide tracks for the transport. The dynamic co-localization of membrane trafficking compartments and individual cytoskeleton filaments is difficult to resolve with current live cell imaging technologies. It is known however, that the Golgi network is located around the centrosome [29]–[31], that the Golgi apparatus has a certain influence on the microtubule network [32], and changes or even interferes with the cell cycle [31], [33], [34]. The ER is linked to the Golgi via microtubules [35]–[38], and the ER-Golgi complex is the principal secretory unit [39]. Also endocytic carrier vesicles follow microtubules [10], [40], [41].

The microtubule network provides a centred structure (see Figure 4). In a reasonable network, central compartments should be found in the centre of the structure. This is true for the Golgi stacks [30]. Accordingly, the Golgi is the central compartment of the membrane trafficking network not only regarding its function but also its location. The ER, where all proteins are translated, is tightly and directly connected to the Golgi via microtubules [38]. Likewise the sink of the secretory pathway should be in a central position, and indeed late endosomes and lysosomes are reported to be in a perinuclear region [3], [42]. The challenge for future work is to explain how vesicles are sorted to the corresponding tracks.

Cytoskeleton connections also can provide the location for the creation of new compartments, for instance the connections between the ER and the Golgi as well as on the actin cables in yeast endo- and exocytosis [43]. Diffusing vesicles first ‘condensate’ onto the cable, then walk along it and subsequently form an endosome to which further vesicles can fuse. The same holds for the ER-Golgi intermediate compartments (ERGICS) found between the ER and Golgi [3].

Additionally, the cytoskeleton can regulate the access to certain compartments, simply by blocking the way. The two main compartments of the simulations were caged in the cytoskeleton network and could not diffuse away. Likewise the probability of an unintentional fusion with large compartments can be reduced. For instance, it is highly likely to reach the relatively large plasma membrane by diffusion because it is evenly distributed around the cell. In order to reduce the collision probability, access to the plasma membrane might be restricted by the actin network. Since actin mainly polymerizes along the plasma membrane, vesicles that are too large to pass through its meshes cannot reach the plasma membrane. Still, the actin network could catch and store vesicles close to the surface until they are needed, for instance at a synapse. Any regulation of the actin cytoskeleton e.g. by Ca2+ can accordingly control vesicle fusion at the plasma membrane [27], [44], [45].

### Dynamics and Dependencies of the Vesicle Model

#### Budding Process and Recycling of Coat Molecules

**Figure 5:**
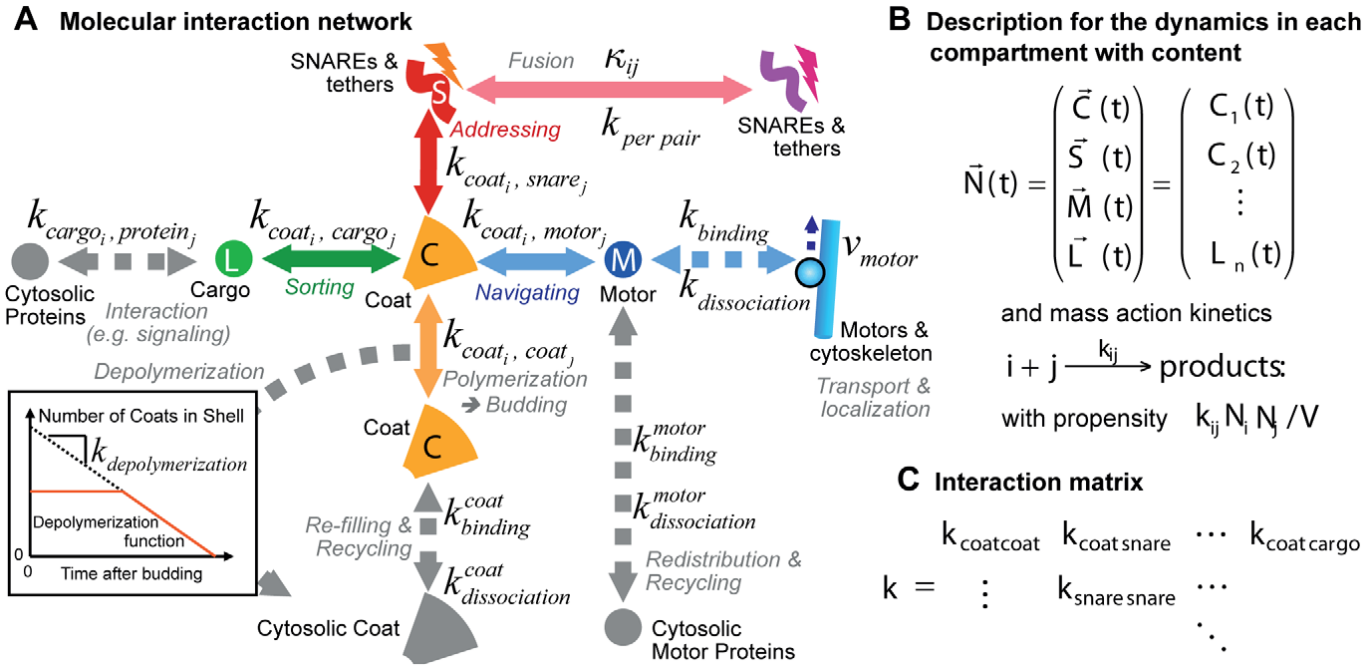
Mathematical Description. A: Interactions and rate constants between the molecules of the vesicle machinery function (cf. Table S1 for a description of the molecule species in the present model). Note that all rate constants are actually a matrix where the number of lines/columns depends on the number of coat/snare/motor/cargo-species as indicated in B and C. The coloured species are bound to the vesicle, while the grey species are located in the cytoplasm. This figure also shows the interaction with cytosolic proteins (e.g. the activation of signalling molecules by endocytosed receptors), as well as the binding/dissociation of cytosolic coats and motors to the vesicle surfaces. The subfigure exemplifies the simplified depolymerization function used to describe the degradation of the coat shell upon budding.

**Figure 6:**
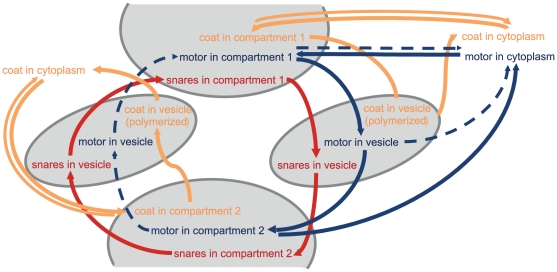
Recycling. Coat molecules are recycled via a cytoplasmic pool of unbound molecules. Motor proteins can be recycled via recycling vesicles or a cytoplasmic pool. Snares can only be recycled via recycling vesicles. Figure S1 shows this process in the data of a simulation.

The vesicle budding process (mediated by the formation of the corresponding coat-shell) crucially depends on the abundance of coat molecules at the budding site (cf. Equation (9) in the Methods section, Figure 1c and Figure 5). Once the vesicle is formed, the respective coat molecules are transported away together with the vesicle. If new (recycled) coat molecules do not replace them, the budding site will be quickly depleted [46]. Figure 6 shows how coat molecules cycle between a cytosolic pool and the compartment membrane.

In vivo, the binding depends on the lipid composition of the membrane and further subspecies of the coat [3], [46]. This is accounted for by adding generic ‘coat catching molecules’ to our model, while Heinrich and Rapoport [12] only included it by the sole affinity of the coats for a compartment. In our model the ‘coat catching molecules’ (index 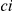) are bound to the compartment (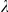) and trigger the association of cytosolic coat molecules 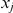 with the compartment with the rate constant 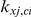. Since a compartment (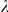) can contain several ‘catchers’, the binding rate constant for a coat molecule to the compartment (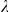) is given by 
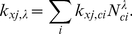
(1)


The catchers are just a special part of the vesicle cargo molecules in our model (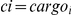). The different compartments should have different characteristics and as such different ‘catching molecules’. This leads to a preference for different coats. Since the coats themselves show a preference for different cargo, SNARE, and motor proteins (cf. Figure 5 and Equation (10) in the Methods section), thus the selectivity and directionality of the transport is established.

In the present discrete framework every coat-polymerization event is covered. As such also every budding event can be observed from the coat concentration in the donor membrane (cf. Figure S1). In order to have similar conditions for subsequent budding events, the recovery rate of coat monomers in the membrane should be large enough to restore the initial conditions prior to the next event. It can be concluded from this fact, that the turnover between membrane bound and free coat molecules should be high, i.e. large binding and dissociation rates as reported by [47]. Also, a sufficient number of free coat molecules in the cytosol is needed. This conclusion is supported by the findings of Forster et al. [24] reporting a cytosolic fraction of about 50%. **In the present model therefore a high coat binding and unbinding rate constant is specified (see Text S2)**.

#### Recycling of SNAREs and Motor Proteins

As shown in Figure 6 motor proteins can also be recycled through the cytosol [48]. In contrast to coat molecules the dissociation rate has to be slow for motor proteins. Otherwise vesicles that need them would lose them too quickly. Accordingly the binding rate has to be slow as well, so that a reasonable steady state can evolve in each compartment. **Therefore a low motor binding and unbinding rate constant is specified in the present model (see Text S2)**.

Here: (i) donor compartment bound motors are incorporated into the vesicle during the budding process, (ii) then they transport the vesicle to the target compartment, to which they are integrated, (iii) from there they slowly dissolve into the cytosol, (iv) eventually diffuse back to the donor compartment, and (v) bind to ‘motor catching proteins’. Thus the presence of motors in each donor compartment depends on its ‘motor catching protein’ settings. The following consideration shows, why motor proteins cannot be recycled in a vesicle bound stage. Assume that motors could be transported back on vesicles going into the reverse direction. Then the vesicle must carry less motors of the kind that has to be recycled than the motors needed for their own transportation. Otherwise the carrier motors would most likely lose the tug of war between both kinds of motors and the vesicle is transported back to its donor compartment - or more correctly: it would never leave it. Thus the total number of motors which is needed for the vesicle transport between two compartments can only be recycled either in an at least partly inactive state via vesicles or via diffusion through the cytosol.

SNARE proteins in contrast cannot diffuse through the cytosol. If they did, the compartment identity would diffuse away with them. Since SNAREs cannot be created in every compartment, they have to be recycled or otherwise be transported towards the compartment where they are needed. Such SNAREs that are only transported - but are not meant to be the SNAREs determining the target of the vesicle - still interfere with the vesicle addressing process. In the two-compartment model introduced by Heinrich and Rapoport [12] therefore 98% of the recycling vesicles fuse with their donor compartments; only 2% reach their target. If the spatial aspects are included, the backward fusion rate will be even higher because the vesicles are initially much closer to the donor compartment, which results in an increased fusion probability.

As soon as the directed transport with motor proteins is included in the model, the recycling of SNAREs becomes much easier. Motor proteins now determine the direction. SNAREs in a vesicle will only interact with SNAREs of the compartment to which they are transported by motor proteins. Provided that the motorized transport worked correctly, the recycled SNAREs are just an additional cargo of the vesicle. Figure S1 shows how a stationary SNARE distribution develops in a two-compartment system. **Since the correct targeting of the vesicles evolves out of the transport with motor proteins along the cytoskeleton, the cell does not need a separate set of t- and v-SNAREs for every transport route**. SNAREs can be used for several connections, which is in agreement with the current knowledge for instance in yeast vesicle transport [49].

### Properties of the Model

The fine-tuning of the parameters and interactions shows interesting properties of the vesicle transport process. It is worth noting, that the effects of SNARE triples, Rabs, GTPases, which further modify the specificity of the process are implicitly accounted for by the effective interaction parameters of the generic coat, SNARE, and cargo species of our model. The following points highlight how the functionality and efficiency of vesicle transport can be assured therein.

#### Vesicle Fusion and SNARE-Interaction

The description of the vesicle fusion based on matching SNARE combinations implies that nonmatching vesicles (those that belong to a different transport module) will actually bounce off of the nontarget compartment. However, there are no reports discussing or showing these rejected vesicles. The absence of reports describing such bounced vesicles can lead to two conclusions: (i) this effect has not yet been investigated, or (ii) the vesicle sorting and transport machinery is efficient enough to lead every vesicle to its correct target. Then however the SNARE interaction is not relevant for the targeting process, because the target selection occurs at an earlier stage.

In the present simulation, in turn, vesicles were bounced frequently - even matching pairs might need several collisions until they finally fuse together. This suggests that the actual fusion of vesicles has to be observed with a greater spatial and temporal resolution in future experiments. Based on more detailed studies of vesicle paths the vesicle model then needs further adjustments.

#### Influence of the Coat on the Backward Fusion Probability

Since the recycling of the SNAREs has to occur alongside with the regular vesicle transport [12], the reduction of the backward fusion probability is of great importance for the functioning of the vesicle transport system. One possibility to achieve this goal comes from the coat that formed the vesicles. As shown in Figure 1c, the coat around the vesicle breaks apart from it during the transport process. However, the coat shell could as well shield it against fusion events. If the depolymerization process of the coat starts only once the vesicle is far enough away from the donor compartment, the backward fusion probability is reduced.

In our simulation we varied the speed of the coat depolymerization process (cf. Figure 5) and indeed the forward to backward fusion ratio increased when the depolymerization is slower. In the present simulation vesicles can only fuse if their coat is completely depolymerized. Recent experimental findings indicate that the coat actually dissociates only after tethering with the target compartment [50].

The clathrin coat, which mediates endocytosis at the plasma membrane, provides an even stronger way of preventing a back-fusion: the involved actin polymerization leads to an actin boost that pushes the vesicle away from the plasma membrane (cf. Equation (12) [43], [51], [52]. The impact of the actin boost depends on its duration, the resulting transport velocity, and the fraction of the resulting force that is perpendicular to the plasma membrane.

Once the vesicle is far enough away from the plasma membrane, it can employ a diffusive search strategy for the target without an oversize risk of returning to the plasma membrane. Again the simulation shows that the backward fusion probability decreases if the actin boost acts stronger (faster velocity or slower coat depolymerization).

#### Regulation of the Budding Process (Cargo Dependent Budding On Demand)

The cargo molecules in the donor compartment can control the vesicle formation process by regulating the turnover of coat molecules [24], [53]. This makes sense because otherwise the cell would form many empty yet costly vesicles. Two properties in the present model account for this fact:

1. the probability for a budding event depends on the cargo concentration in the donor compartment. A cargo-coat-dimer, formed based on Equation (10), initiates the budding event in the simulation. If no cargo is present, no vesicles will be formed.
2. the number of cargo molecules that are incorporated into a vesicle depends on the cargo concentration and the budding time as described above. The more cargo molecules are present, the more cargo can bind to the coat molecules - up to a saturation level.

Thus the cargo flux depends on the cargo level in the donor compartment. This feature will also be discussed in the example on receptor-mediated endocytosis, where it is employed to regulate signal transduction.

### Summary of the Model: Dynamics and Space are Intertwined

1. The functionality of the model has to be established based on the modular coat-SNARE-etc.-machinery. In the corresponding interaction matrix independent sub-matrices govern the interaction of each module (cf. Figure 5), e.g.
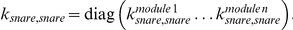

2. Despite the uncertainty of the parameters, the given model is in agreement with the findings of Presley et al. [47] and Forster et al. [24] in stating that a fast exchange between the cytoplasmic and membrane bound pool of coat molecules is needed. In addition, it predicts that motor proteins are recycled via a cytoplasmic pool as well and that the exchange between the pools has to be slow. Furthermore it highlights the role of coat molecules in the prevention of back-fusion events.
3. In a 3D model, also the functionality of the model regarding the targeted translocation of the vesicles through the (structured) intracellular space is important.
4. Obviously both aspects are intertwined because the (local chemical interaction) fusion machinery can only be tested given that the physical transport of the vesicle is working correctly. Since transport with motor proteins is assumed, the (chemical) loading of motor proteins during vesicle budding yet requires a functional vesicle machinery. This also requires that the concentrations of the molecules of the vesicle machinery remain constant. The maintenance of a functional system can be established by recycling of the components. Coat, SNARE, and motor proteins are recycled on different routes as shown in Figure 6.

### Application: Receptor mediated endocytosis and cell polarization in signal transduction

The output of each signal transduction module is naturally determined by the input. As such the number of receptor molecules regulates signal transduction at the most prominent position. More active receptors lead to a stronger signal. The receptor number, in turn, is governed by the degradation and assembly rate of receptors. Both steps occur in a membrane trafficking pathway: (i) the secretory pathway from the ER via the Golgi to the plasma membrane, and (ii) endocytosis via endosomes towards the lysosome. A stationary number of receptors is reached if both rates are balanced. The activated receptor with a bound ligand is able to trigger its endocytosis [54]. Receptor mediated endocytosis is well explored for example for the Ste2p receptor activated by the mating pheromone 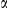-factor in the yeast *Saccharomyces cerevisiae* [55], [56].

**Figure 7:**
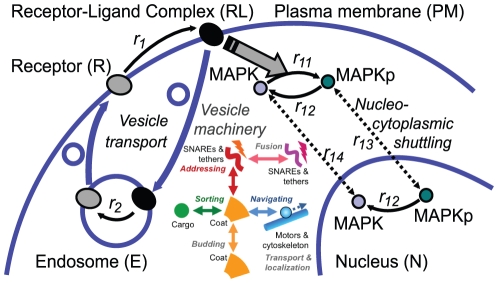
Reduced Model of Receptor Mediated Endocytosis Coupled with Signalling. The signalling cascade is reduced to one stage for simplicity. Endocytosis is driven by the molecular interactions of the vesicle machinery, i.e. Coat, SNARE, cargo (here the receptors), and motor molecules as indicated in the subfigure. The parameters are given in Additional material, Text S2.

Figure 7 shows the setup of the present model, the parameters of the signalling module are given in Table 1 and the parameters of the vesicle system in Text S2. The manifold of membrane trafficking compartments is reduced to one endosome in a central position of the cytoskeleton, next to the nucleus. Vesicles can transport receptors from the endosome to the plasma membrane and the active receptor ligand complex from the plasma membrane to the endosome.

**Table 1:** Rate Constants for the Combined Model.

r	Description	Kinetics/Propensity	Rate Constant
1	**R** 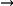 **RL**	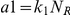	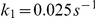
2	**RL** 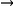 **R** **(only in endosome)**	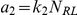	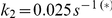
…	**vesicle machinery**		see Text S2
11	**MAPK+RL** 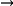 **MAPKp+RL**	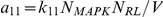	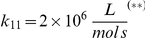
12	**MAPKp** 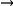 **MAPK** **(in cytoplasm)**	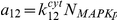	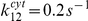
12	**MAPKp** 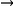 **MAPK** **(in nucleus)**	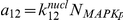	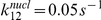
13	**MAPKp** 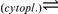 **MAPKp** 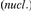	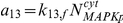 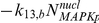	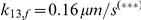 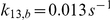
14	**MAPK** 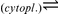 **MAPK** 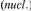	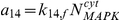 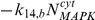	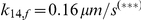 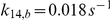

Definitions and rate constants of the signal transduction process. (*) For modelling purposes 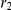 is constituted as 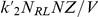 where 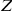 is an enzyme with concentration 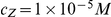 (394 molecules in the endosome) and 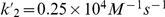. (**) The activation of the signalling molecule MAPK by the receptor ligand complex RL can be triggered by RL molecules either in the plasma membrane, in endocytic vesicles or in the endosome. The activation rate constant of RL in vesicles and endosomes is set to 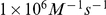. (***) Binding rate to the surface of the nucleus (with concentration given in 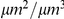 with respect to the volume of the cytoplasm, modelled in analogy to Equation (5a) as described in Methods). The diffusion coefficient of the signalling molecules is set to 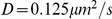.

**Figure 8:**
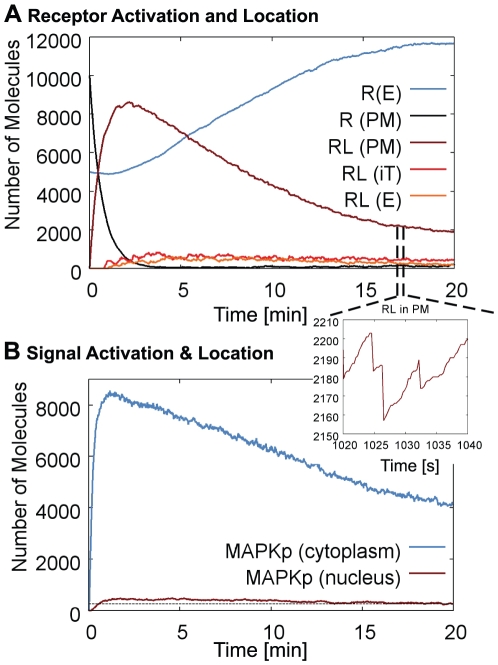
Combined Membrane Trafficking and Signaling Dynamics. (a) The receptors (R) are activated by the binding of the ligand (RL = receptor ligand complex) and subsequently transported from the plasma membrane (PM) via transport vesicles (iT = in transit) to the endosome (E). The excerpt of the receptor ligand complex (RL) on the right shows single budding events from which the budding frequency and the cargo load of the endocytic vesicles can be derived. (RL) is deactivated in the endosome. Therefore the number of inactive receptors (R) in (E) is increased. The number of active receptor ligand complexes (RL) is reduced due to the endocytosis and deactivation reactions. (b) Accordingly, also the number of active MAPKp signalling molecules is reduced.

At 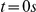 the ligand is added, leading to a fast activation of nearly all receptors (R) in the plasma membrane by the formation of the receptor-ligand (RL) complex (see Figure 8). This triggers endocytosis, which reduces the number of active receptors in the plasma membrane. The receptors are quickly deactivated in the endosome and partly recycle back to the plasma membrane. Due to the time for the budding and transport process, the receptors only arrive with a delay in the endosome. The budding process at the plasma membrane and the endosome depends on the number of receptors as described in the previous section. Thus the increased number of receptors in the endosome increases the recycling flux to the plasma membrane while the endocytic flux is reduced due to the lower number of active receptors. Finally, a steady state is reached. The excerpt in Figure 8 shows random budding events and the varying cargo load of the vesicles, which lead to an irregular sawtooth shape. The strong fluctuations in the receptor number fundamentally differ from the stable number obtained from differential equations or the normal fluctuations in stochastic models. The flux of receptors can be adjusted (i) by the number of receptors transported in a vesicle (up to a saturation limit) and (ii) by the budding frequency of the vesicles and hence the number of vesicles. It is also worth noting, that the present model preserves the delays of the endocytosis and vesicle transport process.

Figure 8 also shows, that the number of active MAPKp (here Fus3) signalling molecules (both in the cytoplasm and the nucleus) is equally reduced when the number of active receptors is reduced. The active receptors accumulate in the endosome. In the present model, the MAPK molecules can be activated there as well. However, the number of active receptors in the ‘signalling endosome’ is rather small (due to the fast deactivation of the receptors). At least with the present parameters, the remaining active receptors in the endosome do not increase the signalling output although they are closer to the nucleus. The present model can accordingly be used to test the signalling endosome hypothesis for different cells and signalling pathways [54], [57].

**Figure 9:**
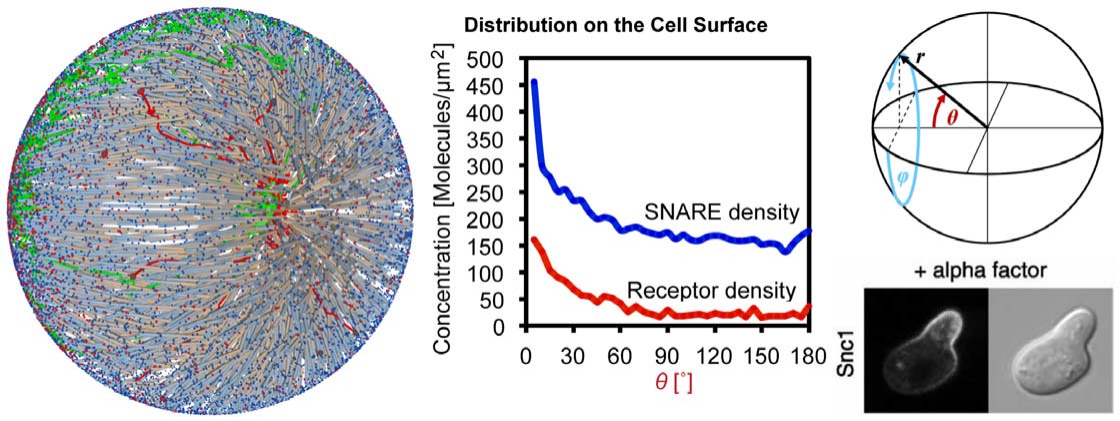
Cell Polarization. Molecules, vesicle paths and cytoskeleton structure. SNAREs (blue) and Receptors (red) accumulate on the left. Endocytic vesicle tracks are shown in red, recycling paths in green. The polarization of the cell is in agreement with the findings of Valdez-Taubas and Pelham [58] for the SNARE Snc1. The microscope image is reprinted from (Valdez-Taubas and Pelham, 2003) with permission from Elsevier.

It is worth noting, that signal transduction can trigger the polarization of the cell, leading to the formation of a mating projection [56], [58], [59]. Figure 9 shows the model cell and the path of the transport vesicles. Due to the slightly polarized shape of the cytoskeleton, exocytic vesicles tend to arrive more at the left of the cell, leading to an accumulation of receptors and SNAREs on the left. The slow diffusion in the plasma membrane preserves their polarized distribution. This is in agreement with the findings of Valdez-Taubas and Pelham [58]. Receptors that are redistributed by this endocytic cycling process increase the signal and the polarization of the cell in a stabilizing feedback loop.

### Conclusions and Outlook

The present model is the first vesicle transport model tracking individual (agent-based) vesicles through the complete cell where the actions (budding,fusion) are determined by the molecular content of the vesicles in a multi-scale manner. This first step towards a systems-oriented understanding of vesicle transport includes the vesicles and compartments and the molecular vesicle machinery. This has the advantage that the control of the molecular interactions on the sorting and transport events can be explored and that the model can be coupled with other molecular processes like signalling. Eventually, such a multi-scale model might be able bridge the gap from molecular interactions to cellular phenotypes.

The modular structure and the sequential actions of vesicle transport nevertheless allowed the parameterization of the model, which led to a functional setup. The constraints of the possible parameter sets also revealed further principles of vesicle transport: the true function can be worked out in a process of elimination of models and parameter sets that do not lead to functional vesicle transport connections. So far, the present vesicle model is able to reproduce budding, transport, and fusion events. The time constants of the reactions and transport processes are only set relatively to each other and do not necessarily match with reality. This is owing to the fact that present experimental results mainly focus on the functional and qualitative identification of molecular interactions and vesicle pathways and not yet on the dynamics of the system. With further spatiotemporal data the model can not only be better adjusted but also include additional molecule classes for a fine tuning of the sorting and fusion process.

For instance a more realistic cytoskeleton for the simulation can be derived from microscopy images. Advanced live cell imaging techniques are required to extract cytoskeleton information and vesicle tracks together in order to resolve the vesicle-cytoskeleton interplay. The current model has to be repeatedly tested against such experimental results and improved in an iterative, data driven way, for instance by comparing the visualized results with life cell images until the model returns the right phenotype of compartment distributions. Altogether, the advances in modelling and visualization techniques provide a tool to investigate the functionality and characteristics that emerge out of the nontrivial interactions in complex systems [60].

Finally, the definition of the identity or type of compartments is another challenge in the dynamic vesicle network. After all, the membrane trafficking network consists of many subtypes of compartments, which dynamically develop, change, or maturate. The identity of the compartment can be related to its content. Alternatively it could be related to the history of the compartments which themselves then depends on the history of their donor compartments (and so on). Both, the event based simulation algorithm and imaging technologies tracking individual vesicles can record the history of every compartment. A detailed map of budding and fusion events can then be extracted from these records in order to relate both concepts of the compartment identity, thus connecting function, content, and lineage of the vesicles.

## Methods

The vesicle model as outlined above and in Figures 1 and 4 is based on distinct molecule classes (coat, snare, motor, and cargo), which are described in Table S1. The vesicle model will be described below and it is embedded in an agent-based simulation method [61] extending the actions of those agents that represent vesicles or compartments.

The general model consists of a set of molecule species and their interactions in a cell (cf. Text S2). Each molecule of each molecular species is tracked individually in the simulation (state variable 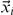 for each molecule 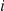). Starting from the initial distribution, the agent-based simulation propagates the agents through space and time. Thus the simulation returns the particle numbers 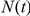 of each species and the molecular distribution in space and time, which can be visualized in various ways [60], [62].

### General Agent-Based Framework

In the agent-based description of the transport and reaction processes of proteins and vesicles, each instantiation of a molecule is represented by a molecule agent at the corresponding position 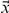 and inherits its properties from the molecule species in an object oriented way. One agent is created for every molecule of the model. Each vesicle agent (representing a vesicle or compartment) is instantiated based on the definitions of the initial compartment it represents or based on the budding reaction in which it was created. The spherical agents with the size of the respective molecule/vesicle move in a discrete time (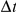) continuous space random walk [63]

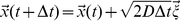
(2)with diffusion coefficient 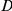 and random number 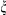 with mean 0 and variance 1 in order to mimic the diffusion process. Alternatively they directly follow a cytoskeleton filament (unit direction 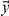) with the speed 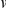 of the motor protein [62]:
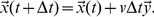
(3)


Reactions in the model are based on mass action kinetics. Two agents will in principle react with each other in a **bimolecular, second order reaction** if a reaction with rate constant 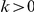 is specified between them and if they are within the reaction volume [61], [64]

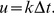
(4)


Note that the molecules first have to meet before they can react. If the agents are not allowed to overlap, the reaction volume is wrapped around them as a thin reaction layer and the two agents 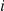 (with radius 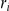) and 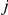 (with radius 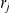) will react if the current distance is smaller than
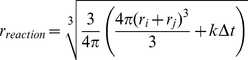
(5a)If the molecules are modelled such that they can overlap, the collision distance
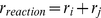
(5b)is used as a critical distance between the molecules. In the latter case, the corresponding interaction volume 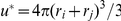 is matched with the reaction volume of Equation (4) by introducing the reaction probability
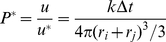
(6)i.e. the reaction between the two molecules will be executed if they are closer than 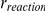 and if a uniform random number 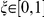 is smaller than 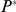. Klann et al. [61] verified this approach and show how this description can be refined in order to take into account the diffusion-limited nature of bimolecular reactions, which is also implemented for the vesicle simulator. Binding to a cytoskeleton filament for directed transport with motor proteins and binding/association with the plasma membrane are also second order reactions (between agent and structure) and evaluated by the same distance-dependent principle [61]. Since the agents must not overlap with the structure, the formalism of Equation (5a) has to be used, however based on the respective geometrical properties of the obstacle.

In contrast, **unimolecular, first order reactions** describe processes, which (on the modelling level) do not depend on molecular interactions and occur spontaneous independent of the position (like for instance a molecular decay). If a first order reaction is assigned to a molecule species, any agent representing a molecule of that species will undergo this first order reaction in the current time step with probability [65]

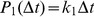
(7)which is again tested by comparing the probability with a uniform random number.

**The full algorithm is accordingly:**

1. Initiate the simulation, load molecules and reactions, setup the cell.
2. Increase the time by 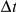.
3. Move the agents according to Equation (2) or (3) depending on the state of the agent (where steps into an obstacle are rejected).
4. Check for second order reactions between two agents or an agent and a cellular structure. A pair will react if they are closer than the corresponding 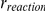 (Equation (5a/b)) and in case of (5b) also if a random number is smaller than 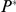 (Equation (6)).
5. Check for first order reactions, which occur independent of the agents position with probability 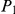 (Equation (7)), i.e. if a random number is smaller than 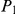.
6. For vesicle agents also check for vesicle actions (see below). If not yet finished, go to 2.

### Vesicle Model

Vesicle agents represent both membrane enclosed compartments and the transport vesicles that are exchanged between the compartments. I.e. compartments are just large vesicles. Vesicle agents are modelled as non-overlapping spheres (except for fusion and budding events, i.e. when they merge/divide). Their diffusion coefficient is calculated based on the Stokes-Einstein relation 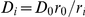 relative to a reference object with 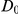 and 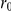. Most vesicle actions are driven by molecular interactions between the molecules of the vesicle machinery (cf. Figure 1, Figure 5, and Table S1).

This vesicle machinery runs within each vesicle/vesicle agent in a multi-scale manner. The present model assumes well-mixed conditions inside the vesicles. Thus it is sufficient to track the number of molecules 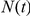 of each species, the positions inside the vesicle are not necessary. In order to track the vesicle state and the reactions occurring in the vesicles, the following information is stored for each vesicle/compartment:

- **Identity:** The type of the agent identifies the compartment e.g. as Golgi. Furthermore its donor compartment is stored to resolve its origin.
- **Cargo:** Vesicles/compartments contain different molecules. These molecules are further categorized in six groups (three of them have already been defined by Heinrich and Rapoport [12]):
  - monomeric Coat molecules that are bound to the vesicle surface.
  - Coat molecules that are in the polymerized state (in the budding process).
  - SNARE proteins (which can promote vesicle fusion).
  - motor proteins for the transport along the cytoskeleton.
  - membrane bound cargo molecules (e.g. receptors). (where the mass action kinetics based reaction propensity is calculated based on the surface area)
  - cargo molecules that are located inside of the vesicle (where the mass action kinetics based reaction propensity is calculated based on the volume).
- **Volume, Surface, Radius:** All three parameters are tracked separately for the following reason. Both, the vesicle surface area (lipids) and the vesicle volume are conserved in all processes. Due to the different exponents of volume and surface for a sphere, both numbers are not in agreement after a fusion event. Still the vesicle agents are modelled as spheres, which radius is calculated based on the volume. The surplus-surface could be arranged e.g. in a wavy, corrugated manner around the sphere. Future work could track other compartment shapes as well to account for the effects of different surface to volume ratios.

The plasma membrane itself also constitutes a compartment, from which vesicles can be formed in the endocytosis process, and to which exocytic vesicles can fuse. Since the plasma membrane corresponds to the particle based simulation framework, all ‘cargo’ molecules of the plasma membrane have to be modelled explicitly as particle agents. A special interface treats the import of these molecules into endocytic vesicles.

### Vesicle Actions and Reactions (Inside the Vesicles)

The internal reactions are based on mass action kinetics. Since some molecules of the vesicle machinery are of low abundance, we use a stochastic integration scheme. In the well-mixed spatially homogeneous environment of the vesicle/compartment, the propensity 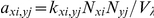 has to be evaluated, where the indices 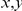 correspond to the class of coat, snare, or cargo proteins and 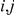 define the molecule species within that class (cf. Figure 5). Additional indices 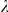 can define the location/compartment respectively. The total probability of a reaction in 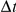 is 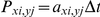 (to first order in 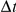, in analogy to Equation (7)). The probability of any individual 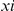 molecule to react in this reaction is then 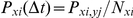 or directly:
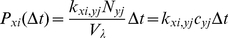
(8)(note the exchangeability between 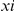 and 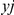). All 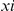 molecules in the compartment are tested for the reaction (i.e. they will react if a uniform random number is smaller than 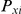). The probability 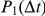 for first order reactions of each molecule in the vesicle is calculated for every time step as described in Equation (7). If a reaction happens, all concentrations (and subsequently also the reaction probabilities) are updated.

We acknowledge that the Gillespie stochastic simulation algorithm (SSA) [66] provides a fast way to simulate the stochastic reaction process. Therein the time to the next reaction is sampled from an exponential distribution based on the uniform random number 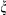:
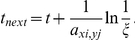
Therefore just one random number is needed per reaction, while on average 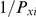 random numbers are needed in our approach. However the SSA assumes a closed system, while vesicles can exchange molecules with the environment and fuse together within 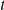 and 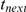 in a process that is driven from outside the SSA system.

#### Reactions of the vesicle cargo

Cargo molecules can interact with each other if a reaction is specified. For example they can be degraded or processed in the vesicles. Cargo reaction events are evaluated based on Equation (7) for first order reactions and Equation (8) for second order reactions. The discrimination between membrane bound (v) and luminal (vi) molecules is necessary because the propensities in the used mass action based kinetics have to be calculated based on the membrane surface or the vesicle volume respectively.

The reactions of the vesicle machinery molecules (i)–(iv) will be explained separately in the following because they govern the budding, transport, and fusion process.

#### Budding Process with Coat Molecules

On the first look, vesicle budding looks like a simple reaction in which new vesicles are created with a specific propensity. However this rate is determined by the respective cargo for the new vesicle (on demand) and furthermore by the availability of the respective machinery molecules: the coats (cf. Figure 1 c). In the simulation, budding is initiated by the formation of a coat-cargo dimer based on the coat(i)-cargo(j) reaction rate constant (cf. Figure 5). The respective probability is given by Equation (8). Then the coat is polymerized until the coat shell is complete (i.e. 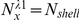) with the propensity
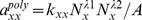
(9)with 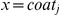, 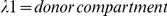, 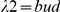, 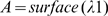 (for the corresponding probability see Equation (8)).

During this time the new vesicle agent is pushed out of the donor compartment until it is completely separated (the time it took is stored in 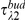). Only at this time point the *cargo*, *snare*, and *motor* proteins are transferred to the vesicle based on the corresponding rate constants (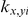 with 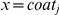, 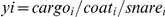 cf. Figure 5) and the propensities
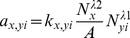
(10)


Due to this approach the reaction probability has to be calculated over the whole budding time 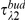, where a constant cargo number and the average of the polymerized coat shell (i.e. 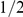 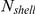) is assumed. The probability for each cargo molecule (Equation (8)) must accordingly be calculated as
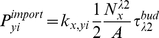
(11)


This approach is justified as long as the numbers of the cargo molecules vary much slower than 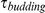. Additionally the saturation of the import is included in the following way: cargo, snare, and motor proteins are only transferred into the vesicle up to a predefined maximal number (for instance because the transmembrane domains of the coat can only bind a limited number of cargo molecules). Since all molecules of each class (snare, motor, or cargo) compete in this process with all other molecules of their class, the following algorithm is used:

Loop: for all classes (snare, motor, or cargo)

1. For each species of that class. Calculate a try number of molecules that will be transferred into the vesicle.
2. If the sum of these try numbers exceeds the limit of the class, then all numbers in that class are multiplied with the factor 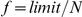 such that the final sum does not exceed the 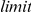.
3. This modified number (including the saturation) of molecules is transferred into the new vesicle.

After the budding event, the coat starts to depolymerize based on a predefined depolymerization function (see Figure 5).

#### Endocytosis

Vesicles can also bud from the plasma membrane in the endocytosis process (mediated by the clathrin coat). In the plasma membrane all molecules are modelled explicitly. The coat polymerization process is therefor modelled as clustering of membrane bound coat molecules. Likewise *cargo*, *snare*, and *motor* proteins bind to these clusters based on the respective reaction rate constants. The critical binding distance (cf. Equation (5b)) is depending on the rate constant and the number of coats that are already bound to the cluster).

Once the coat cluster has reached the required size 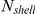, a new vesicle agent is created at the budding site and all *coat*, *cargo*, *snare*, and *motor* proteins are transferred into it. The vesicle is pushed away from the plasma membrane with the velocity of the actin boost (value given in Text S2) [43], [51], [52]. In the present model the vesicle moves perpendicular to the plasma with the velocity 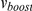. While the coat shell (tracked with 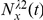) depolymerizes, the random walk of the undirected diffusion takes over:
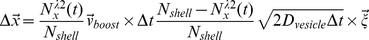
(12)


**Figure 10:**
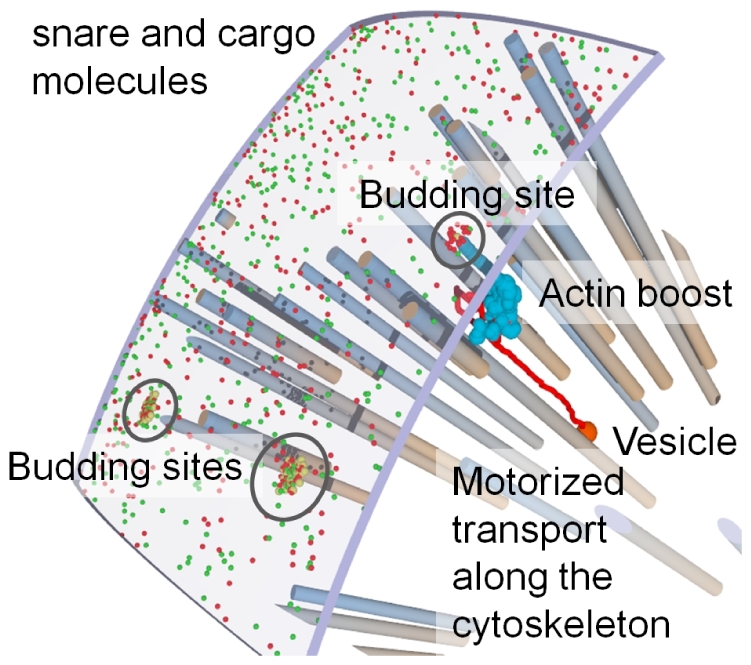
Endocytosis Process in the Simulation: visualized at a section from the plasma membrane. Coat (yellow), snare (green), and cargo (red, here a membrane bound receptor) molecules cluster together and eventually form a vesicle (large red sphere). This is pushed into the cell by the actin boost (path shown in light blue) and can subsequently bind to a cytoskeleton for transport with motor proteins (path during diffusion and motor protein transport is shown in red).

Figure 10 shows the clustering process, the directed path due to the actin boost, a diffusive search until the vesicle finds a cytoskeleton filament, and finally directed transport with motor proteins along that filament into the cell.

### Interactions between Vesicle Agents or other Objects

#### Vesicle Fusion

From the modelling perspective, vesicle fusion is simply a bimolecular reaction between two agents (now again in space: first they have to meet before they can interact). The critical interaction distance (cf. Equation (5a)) between the non-overlapping vesicle 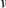 and 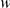 for fusion is calculated based on the fusion rate constant 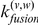. Vesicle fusion depends on the SNARE-interaction as shown in Figure 1b and Figure 5. Therefore the reaction rate constant 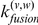 is determined by the SNARE interaction in the following way:

- **SNARE-Interaction:** The SNARE interaction is determined by the preference of the model SNAREs to form a pair. This is encoded in the symmetric matrix 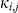 , carrying the interaction strength or preference for each possible SNARE-SNARE-combination [12] (for actual parameters see Text S2).
- **SNARE-Pairs:** The number of SNARE-pairs between vesicle 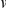 and 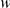 is calculated by multiplying the number of SNAREs of each kind with their pairing strength 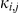:
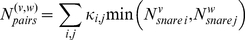
(13)where the SNAREs 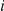 are in compartment 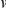 and the SNAREs 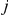 in 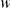. The number of SNAREs is calculated as 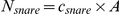 based on the average SNARE concentration (in the membranes) of the vesicles within the interaction area of the vesicles (Text S1). We suggest using the minimal number of both SNAREs because there cannot be more pairs than the smaller number of partners. This differentiates our model from Heinrich and Rapoport [12], who simply multiplied both numbers.
- **Vesicle Fusion Rate Constant:** The rate constant for vesicle fusion is found by multiplying a general fusion rate constant per SNARE-pair 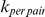 with the effective number of pairs for the respective pair of vesicles:
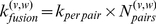
(14)Since vesicle fusion is not an instantaneous process, we similarly calculate the fusion time based on the inverse of the effective number of pairs (more = faster) and a fusion time per pair.

**Attaching to the Cytoskeleton and Motor Protein Transport:** Based on the number of motor proteins in a vesicle (agent), it can bind to the cytoskeleton (reaction volume 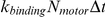). The velocity at which the vesicle moves along the cytoskeleton in Equation (3) is likewise modulated by the number of motor proteins. Motors of different directions lead to a tug of war as explained in Text S1.

**Vesicle-Protein-Interactions:** Likewise cytoplasmic molecules (molecule agents) can interact with membrane bound molecules of the vesicles (for instance active receptor complexes in the vesicle with signalling molecules but also the coat/motor catching process, described by Equation (1)). Based on the mass action kinetics framework the reaction rate constant for each cytoplasmic molecule is obtained by multiplying the specified rate constant with the number of molecules in the vesicle. From this value the critical reaction distance (Equation (5b)) for the reaction between the molecule agent and the vesicle agent is calculated (Note, that vesicle agents and molecule agents can overlap in the present model).

### Parameterization and Performance of the Vesicle Model

First of all, the compartments need to be defined, i.e. their size and the numbers of coat, cargo, snare, and motor proteins have to be declared. Likewise the numbers of free coats and motor proteins in the cytoplasm have to be defined. Based on the desired exchange between the membrane bound and free cytoplasmic pool the respective binding and dissociation rates for coat and motor proteins can then be assigned.

The time for the budding process is determined by the polymerization into the coat shell, i.e. by the coat concentration in the donor compartment and 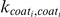. Based on the pre-set coat concentration 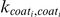 can then be adjusted so that the budding process is accomplished (on average) within the desired time.

The loading of cargo/snare/motor proteins is described by Equation (10). It occurs during the budding process and therefore has to be integrated over the actual budding time. Given that this budding time is close to the desired set point (defined above), the desired cargo/snare/motor concentration in the vesicles is reached by adjusting rate 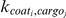, 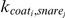, and 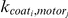 (the cargo/snare/motor concentration in the donor compartment should be close to the initial values, and the average coat concentration in the budding vesicle is given by the parameter 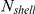 and the standard size of the vesicle [28], [67]. As such, budding leads to vesicles that stochastically vary around the desired set-point of the cargo/SNARE/motor protein numbers in the present stochastic simulation.

Based on the SNARE concentration in the vesicle and in the target compartment finally 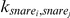 and 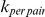 can be adjusted to reach the desired fusion probability in Equation (14).

Recycling of the vesicle machinery compounds as described above is required in order to keep the process at the desired set-point.

The complete model is accordingly given by defining (cf. Text S2)

1. The cell.
2. The molecule species with their properties and (initial) abundances in the cell.
3. The reactions between the molecules: (educt(s), product(s), rate constant.
4. The definition of the vesicle machinery and its interactions (this requires an interface connecting explicitly modelled cytoplasmic molecule species and the molecule species within the vesicle system).
5. The number, position, size, and content of the initial set of compartments and vesicles.

In total up to 100,000 agents have to be tracked in the simulation. (Note, that for performance reasons the total number of cytoplasmic coats and motors can be reduced while keeping the binding rate constant by increasing the binding rate constant in Equation (1)). In order to reduce the computational costs, the step size 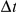 (and 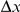 respectively) are set to maximal values of 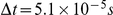 for the set of simulations with 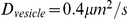 (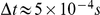 for the slower diffusing set). The random walk is sampled from a uniform distribution instead of a normal distribution but with the same mean and variance which is (i) faster, (b) allows larger 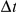 because the distribution does not have long tails which correspond to rare but huge jumps, and (c) within 4 iterations converges to the normal distribution (central limit theorem). Reactions are only sampled every 5 steps, which (a) allows the random walkers to equilibrate and (b) reduces the more costly pair searching of bimolecular reactions. All molecule agents can overlap with each other, but the vesicles are self-exclusive. With these measures our single threaded optimized Fortran simulation, compiled with the Intel Fortran Compiler reached the following performance on a 2×2.4 GHz Quad-Core Intel Xeon Mac Pro with 16 GB 1066 MHz DDR3 memory (while 4 simulations ran in parallel): with 11000 particles and 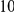 vesicles: 50 s per 10000 iterations, with 100000 particles and 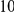 vesicles 320 s per 10000 iterations (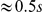 of simulated time). The vesicle routines of the simulation were designed for systems with low vesicle numbers and are not yet fully optimized. The runtime increases by about 60 s at 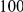 vesicles and grows proportional to 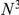.

## Supporting Information

Figure S1 Number and location of one set of molecules of the vesicle machinery: The recycling of SNAREs between the two compartments, i.e. the plasma membrane (PM) and the Endosome (E), is shown in red-black. Coats cycle between the membrane bound and the free cytosolic pool. Due to the rapid exchange the polymerization reduces the number of bound coats only marginally. Also motors (blue concentration profiles) are recycled from the endosome back to the plasma membrane via the cytosolic pool.
(TIF)

Table S1 Description of the vesicle machinery: list of molecule classes in the vesicle transport model. Each class can contain an arbitrary number of molecule species. Note, that vesicles are also compartments by itself. All molecules that bind to a compartment membrane can also bind to vesicle membranes.
(PDF)

Text S1 Additional details of the vesicle model: (a) Vesicle interaction area. (b) Tug of war between motors of opposite direction.
(PDF)

Text S2 Setup and parameters of the vesicle model.
(PDF)
