# Supplementary figures and images for: Spatial Modeling of Vesicle Transport and the Cytoskeleton: The Challenge of Hitting the Right Road
Source: PLoS One. 2012 Jan 12;7(1):e29645. doi: 10.1371/journal.pone.0029645 (PMC3257240; doi:10.1371/journal.pone.0029645)

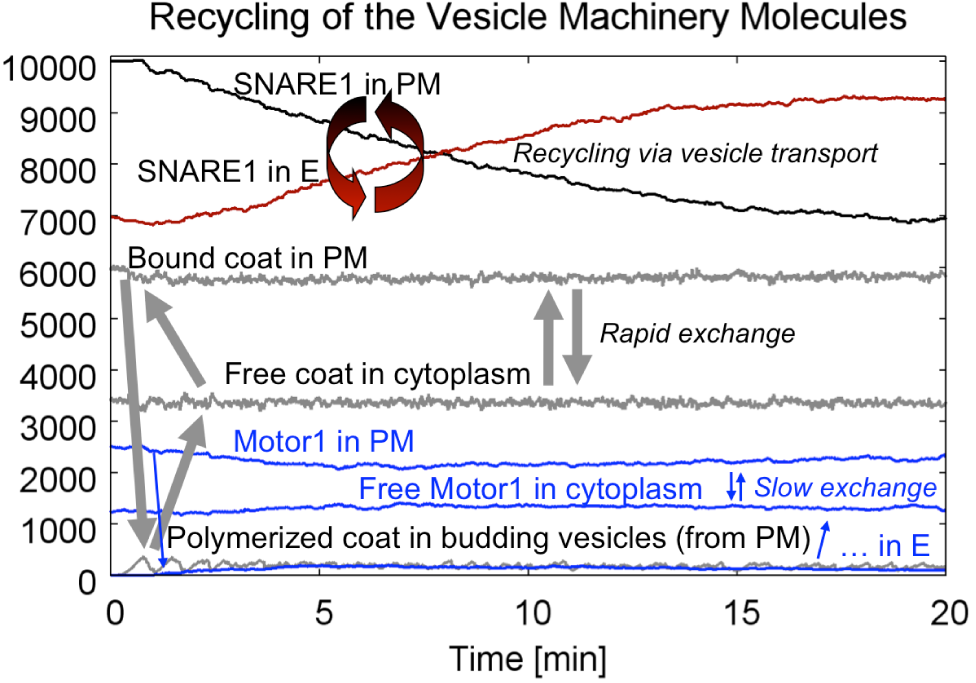

Supplement: Figure S1 — Number and location of one set of molecules of the vesicle machinery: The recycling of SNAREs between the two compartments, i.e. the plasma membrane (PM) and the Endosome (E), is shown in red-black. Coats cycle between the membrane bound and the free cytosolic pool. Due to the rapid exchange the polymerization reduces the number of bound coats only marginally. Also motors (blue concentration profiles) are recycled from the endosome back to the plasma membrane via the cytosolic pool. (TIF) [file pone.0029645.s001.tif]
