# Supplementary material for: Spatial Modeling of Vesicle Transport and the Cytoskeleton: The Challenge of Hitting the Right Road
Source: PLoS One. 2012 Jan 12;7(1):e29645. doi: 10.1371/journal.pone.0029645 (PMC3257240; doi:10.1371/journal.pone.0029645)
Supplement: Table S1 — Description of the vesicle machinery: list of molecule classes in the vesicle transport model. Each class can contain an arbitrary number of molecule species. Note, that vesicles are also compartments by itself. All molecules that bind to a compartment membrane can also bind to vesicle membranes. (PDF) [file pone.0029645.s002.pdf]

Table S1: **List of molecule classes in the vesicle transport model. Each class can contain an arbitrary number of molecule species. Note, that vesicles are also compartments by itself. All molecules that bind to a compartment membrane can also bind to vesicle membranes.**

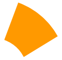

Coat

Function: Vesicle Budding
- Polymerization shapes the vesicle
- Specific interactions select **SNARE, Motor** and **Cargo** molecules to ensure proper sorting

States:
a. free in cytoplasm
b. bound to membrane of compartment
c. polymerized around a budding vesicle

Represents:
COPI, COPII, clathrin

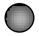

"Coat Catcher"

Function: Selecting Coat Molecules
- Specific interactions bind free **Coat** molecules to the compartment membrane to ensure proper sorting

States:
a. bound to membrane of compartment

Represents:
Specific molecules or motifs in the compartment membrane

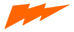

Tethers

Function: Involved in Vesicle Fusion
- Tethering factors tether vesicles, initiating fusion
- Note: not included in the present model, therein their function is taken over by **SNAREs**

States:
a. bound to membrane of compartment

Represents:
(Yeast:) Vps 18, GSG1, Sec34-37, Tip 1, …

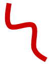

SNAREs

Function: Addressing and Vesicle Fusion
- SNAREs specify the fusion partner ="Addressing"
- SNAREs mediate vesicles fusion

States:
a. bound to membrane of compartment

Represents:
(Yeast:) Ufe1, Sed5, Tlg2, Pep12, Vam3, Sso1, Sso2 Sec20, Bos1, Gos1, Vti1, Sec9, Spo20, Slt1, Sft1, Bet1, Tlg1, Syn8, Vam7, Sec9, Spo20, Sec22, Ykt6, Nyv1, Snc1, Snc2

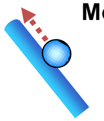

Motors

Function: Directed Transport
- Pull vesicles along cytoskeleton filaments

States:
a. free in cytoplasm
b. bound to membrane of compartment

Represents:
Kinesins, Dyneins, Myosins

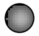

"Motor Catcher"

Function: Selecting Motor Molecules
- Specific interactions bind free **Motor** proteins to the compartment membrane to refill the local pool

States:
a. bound to membrane of compartment

Represents:
Specific molecules or motifs in the compartment membrane

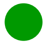

Cargo

Function: … to be transported and sorted
- Cargo molecules are just cargo inside the vesicles
- The presence of Cargo molecules triggers vesicle budding

States:
a. free in the lumen of the compartment
b. bound to membrane of compartment

Represents:
All molecules that have to be sorted and transported in the membrane trafficking system.
Note: **Coat and Motor Catchers** can be treated as Cargo as well in order to transport and sort them.

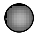

Other Species

Function:
- Other molecules in the cell can interact with the molecules in the compartments

States:
a. outside of the compartments

Represents:
All other molecules in the cell, especially **signaling molecules** that can be activated by membrane bound receptor complexes
