# Supplementary material for: Spatial Modeling of Vesicle Transport and the Cytoskeleton: The Challenge of Hitting the Right Road
Source: PLoS One. 2012 Jan 12;7(1):e29645. doi: 10.1371/journal.pone.0029645 (PMC3257240; doi:10.1371/journal.pone.0029645)
Supplement: Text S1 — Additional details of the vesicle model: (a) Vesicle interaction area. (b) Tug of war between motors of opposite direction. (PDF) [file pone.0029645.s003.pdf]

Text S3: Details of the Vesicle Model

(a) Vesicle interaction area

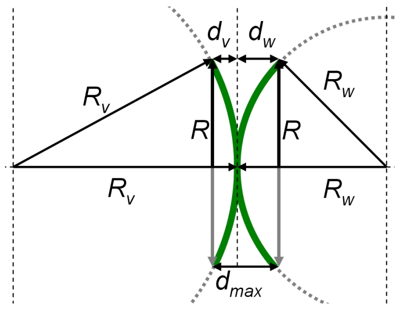

The part of the vesicle surface that is within the maximal distance d_{max} of the SNARE-interaction is highlighted in green. The interacting surface area A is calculated neglecting the curvature of the vesicle (assuming d_{max} << R) as $A = \pi R^2$ with

$$R = \sqrt{\frac{1}{2}(R_v^2 + R_w^2) - \frac{1}{4}(R_v + R_w - d_{max})^2 - \frac{1}{4}\frac{(R_v^2 - R_w^2)^2}{(R_v + R_w - d_{max})^2}}$$

(b) Tug of war between different motor proteins:

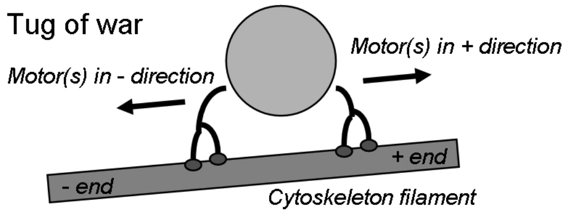

Tug of war between the motors pulling towards the plus and minus direction. This process is mimicked in the simulation by

$$\Delta x = v_0 \times \Delta t \left(\frac{n_{motor+} - n_{motor-}}{n_{motor+} + n_{motor-}} + (\xi_1 - \xi_2) \right)$$

where ξ_1 and ξ_2 are two independent random numbers of the uniform distribution.
