# Supplementary material for: Spatial Modeling of Vesicle Transport and the Cytoskeleton: The Challenge of Hitting the Right Road
Source: PLoS One. 2012 Jan 12;7(1):e29645. doi: 10.1371/journal.pone.0029645 (PMC3257240; doi:10.1371/journal.pone.0029645)
Supplement: Text S2 — Setup and parameters of the vesicle model. (PDF) [file pone.0029645.s004.pdf]

Text S4: Setup and Parameters of the Vesicle Model

The following text files are used to control the simulation and contain the simulation parameters:

- "parameter_input.txt" with the general cell setup, the definition of the particle agents and their interactions. The cytoskeleton structure is however hardcoded in the simulation.
- "Vesicle-Setup.txt" defining the vesicle parameters
  - The Definition of the present vesicle network is as follows:
    - Type 1: Endocytic Vesicles
    - Type 2: Endosome in the endocytosis model
      ER-Compartment in the 2-compartment models
    - Type 3: Golgi-Compartment
    - Type 4: Vesicles Budding from 2
    - Type 5: Vesicles Budding from 3
    - Type 6: all other compartments
- "Vesicle-Particle-Interface.txt" connects the definitions from the vesicle and the particle level for the multi-scale simulation
- "Vesicles-Initial-Compartments.txt" defines the initial vesicle conditions.

Note, that for models that tested vesicle transport only by diffusion the number of motor proteins is set to 0.

Of course we are planning to standardize the input and to define an XML/SBML format in future work …

The simulation is available on request from Michael Klann, mklann@ee.ethz.ch

parameter_input.txt:

```
General Cell Parameters:
5     [x10^-6m] : Diameter of the model cell
49    []        : space discretization for speedup
1 10 100 1000 : Random Number Seed
Particle Parameters:
110000          : Max number of Particles
42              : Number of Particle species
Cytoskeleton Parameters:
10002           : Max number of structures (incl. plasma membrane and nucleus)
45              : Max number of objects in a voxel
0.25   0.6      : relative nuclear diameter, relative displacement from the center along the x-axis (0.4 for the combined vesicle and signaling model)
10000           : Cytoskeleton filaments (0 for the directly connected compartments, 1000 for endocytosis 2-compartment test, 3300 for combined vesicle and signaling model) (diameter: 25 nm)
0.5   [x10^-6m] : Length of the filaments (1.5x10^-6m for endocytosis 2-compartment test, 1.0x10^-6m for combined vesicle and signaling model)
0               : Number of crowding spheres.
Visualization:
0               : Total number of frames
0               : Visualization interval
0.0             : Transparency of the cytoskeleton
Particle Species Definitions:
  *** (1) Dummy Objects ***
1                       : Initial Number
10e-3  [x10^-6m]        : Radius
3                       : Starts in the cytoplasm
3                       : diffuses in the cytoplasm
0.1 [(10^-6m)²/s]       : Diffusion coefficient
0.0 [(10^-6m)/s]        : velocity along cytoskeleton
dummy                   : Name
0.0 0.0 0.              : Color in in RGB
  *** (2) Budding Site Seed in the plasma membrane ***
1                       : (25 for endocytosis 2-compartment test, 15 for combined vesicle and signaling model)
30e-3  [x10^-6m]        : Radius
1                       : Starts in the plasma membrane
0                       : immobile
0.0 [(10^-6m)²/s]       : Diffusion coefficient
0.0 [(10^-6m)/s]        : velocity along cytoskeleton
Ede1p                   : Name
0.0 0.0 0.              : Color in in RGB
  *** (3) Coat 1 - free ***
5713                    : Initial Number
7.5e-3 [x10^-6m]        : Radius
3                       : Starts in the cytoplasm
```

```
3                       : diffuses in the cytoplasm
0.25 [(10^-6m)^2/s]     : Diffusion coefficient
0.0 [(10^-6m)/s]        : velocity along cytoskeleton
Coat_1*                 : Name
0.0 0.0 0.              : Color in in RGB
  *** (4) Coat 2 - free ***
5713                    : Initial Number
7.5e-3 [x10^-6m]        : Radius
3                       : Starts in the cytoplasm
3                       : diffuses in the cytoplasm
0.25 [(10^-6m)^2/s]     : Diffusion coefficient
0.0 [(10^-6m)/s]        : velocity along cytoskeleton
Coat_2*                 : Name
0.0 0.0 0.              : Color in in RGB
  *** (5) Coat 1 - bound ***
0                       : (2500 for endocytosis 2-compartment test, 6000
for combined vesicle and signaling model)
7.5e-3 [x10^-6m]        : Radius
1                       : Starts in the plasma membrane
1                       : diffusing in the pm
0.05 [(10^-6m)^2/s]     : Diffusion coefficient
0.0 [(10^-6m)/s]        : velocity along cytoskeleton
Coat_1=                 : Name
0.0 0.0 0.              : Color in in RGB
  *** (6) Coat 1 - bound in Cluster/Patch ***
0                : Initial Number
7.5e-3 [x10^-6m]        : Radius
1                       : Starts in the plasma membrane
0                       : immobile
0.0 [(10^-6m)^2/s]: Diffusion coefficient
0.0 [(10^-6m)/s]        : velocity along cytoskeleton
clathrin                : Name
1.0 1.0 0.              : Color in in RGB (yellow)
  *** (7) Coat 2 - bound ***
0                       : Initial Number
7.5e-3 [x10^-6m]        : Radius
1                       : Starts in the plasma membrane
1                       : diffusing in the pm
0.05 [(10^-6m)^2/s]: Diffusion coefficient
0.0 [(10^-6m)/s]        : velocity along cytoskeleton
Coat_2=                 : Name
0.0 0.0 0.              : Color in in RGB
  *** (8) Coat 2 - bound in Cluster/Patch ***
0                       : Initial Number
7.5e-3 [x10^-6m]        : Radius
1                       : Starts in the plasma membrane
0                       : immobile
0.0 [(10^-6m)^2/s]: Diffusion coefficient
0.0 [(10^-6m)/s]        : velocity along cytoskeleton
Coat_2=++               : Name
0.0 0.0 0.              : Color in in RGB
  *** (9) SNARE_1 ***
0                       : (20000 for endocytosis 2-compartment test,
10000 for combined vesicle and signaling model)
5.0e-3 [x10^-6m]        : Radius
1                       : Starts in the plasma membrane
1                       : diffuses in the plasma membrane
0.05 [(10^-6m)^2/s]     : Diffusion coefficient
0.0 [(10^-6m)/s]        : velocity along cytoskeleton
SNARE_1                 : Name
0.0 1.0 0.0             : Color in in RGB (green)
  *** (10) SNARE_2 ***
0                       : (48000 for endocytosis 2-compartment test,
12000 for combined vesicle and signaling model)
5.0e-3 [x10^-6m]        : Radius
1                       : Starts in the plasma membrane
1                       : diffuses in the plasma membrane
0.025 [(10^-6m)^2/s] : Diffusion coefficient
0.0 [(10^-6m)/s]        : velocity along cytoskeleton
SNARE_2                 : Name
0.0 0.0 0.0             : Color in in RGB
  *** (11) SNARE_3 ***
0                : Initial Number
5.0e-3 [x10^-6m]        : Radius
1                       : Starts in the plasma membrane
1                       : diffuses in the plasma membrane
0.025 [(10^-6m)^2/s]    : Diffusion coefficient
0.0 [(10^-6m)/s]        : velocity along cytoskeleton
SNARE_3                 : Name
0.0 0.0 0.              : Color in in RGB
  *** (12) SNARE_4 ***
0                : Initial Number
5.0e-3 [x10^-6m]        : Radius
1                       : Starts in the plasma membrane
1                       : diffuses in the plasma membrane
0.025 [(10^-6m)^2/s]    : Diffusion coefficient
0.0 [(10^-6m)/s]        : velocity along cytoskeleton
SNARE_4                 : Name
0.0 0.0 0.              : Color in in RGB
  *** (13) Trans_1 (motorprotein) ***
0                       : (10000 for endocytosis 2-compartment test with
motors, 2500 for combined vesicle and signaling model)
5.0e-3 [x10^-6m]        : Radius
1                       : Starts in the plasma membrane
1                       : diffuses in the plasma membrane
0.01 [(10^-6m)^2/s] : Diffusion coefficient
0.0 [(10^-6m)/s]        : velocity along cytoskeleton
Trans_1                 : Name
0.0 0.0 0.              : Color in in RGB
  *** (14) Trans_2 (motorprotein) ***
0                : Initial Number
5.0e-3 [x10^-6m]        : Radius
```

1 : Starts in the plasma membrane
1 : diffuses in the plasma membrane
0.01 $[(10^{-6}m)^2/s]$: Diffusion coefficient
0.0 $[(10^{-6}m)/s]$: velocity along cytoskeleton
Trans_2 : Name
0.0 0.0 0. : Color in in RGB

*** (15) Trans_3 (motorprotein) ***
0 : Initial Number
5.0e-3 $[x10^{-6}m]$: Radius
1 : Starts in the plasma membrane
1 : diffuses in the plasma membrane
0.01 $[(10^{-6}m)^2/s]$: Diffusion coefficient
0.0 $[(10^{-6}m)/s]$: velocity along cytoskeleton
Trans_3 : Name
0.0 0.0 0. : Color in in RGB

*** (16) Trans_4 (motorprotein) ***
0 : Initial Number
5.0e-3 $[x10^{-6}m]$: Radius
1 : Starts in the plasma membrane
1 : diffuses in the plasma membrane
0.01 $[(10^{-6}m)^2/s]$: Diffusion coefficient
0.0 $[(10^{-6}m)/s]$: velocity along cytoskeleton
Trans_4 : Name
0.0 0.0 0. : Color in in RGB

*** (17) Trans_5 (motorprotein) ***
0 : Initial Number
5.0e-3 $[x10^{-6}m]$: Radius
1 : Starts in the plasma membrane
1 : diffuses in the plasma membrane
0.01 $[(10^{-6}m)^2/s]$: Diffusion coefficient
0.0 $[(10^{-6}m)/s]$: velocity along cytoskeleton
Trans_5 : Name
0.0 0.0 0. : Color in in RGB

*** (18) Trans_6 (motorprotein) ***
0 : Initial Number
5.0e-3 $[x10^{-6}m]$: Radius
1 : Starts in the plasma membrane
1 : diffuses in the plasma membrane
0.01 $[(10^{-6}m)^2/s]$: Diffusion coefficient
0.0 $[(10^{-6}m)/s]$: velocity along cytoskeleton
Trans_6 : Name
0.0 0.0 0. : Color in in RGB

*** (19) Trans_1 (free in cytosol) ***
6855 : (0 for diffusion only, 5750 for endocytosis 2-compartment test and for combined vesicle and signaling model)
5.0e-3 $[x10^{-6}m]$: Radius
3 : Starts in the cytoplasm
3 : diffuses in the cytoplasm
0.1 $[(10^{-6}m)^2/s]$: Diffusion coefficient
0.0 $[(10^{-6}m)/s]$: velocity along cytoskeleton
Trans_1 : Name
0.0 0.0 0. : Color in in RGB

*** (20) Trans_2 (free in cytosol) ***
6855 : (0 for diffusion only, 5750 for endocytosis 2-compartment test and for combined vesicle and signaling model)
5.0e-3 $[x10^{-6}m]$: Radius
3 : Starts in the cytoplasm
3 : diffuses in the cytoplasm
0.1 $[(10^{-6}m)^2/s]$: Diffusion coefficient
0.0 $[(10^{-6}m)/s]$: velocity along cytoskeleton
Trans_2 : Name
0.0 0.0 0. : Color in in RGB

*** (21) Trans_3 (free in cytosol) ***
0 : Initial Number
5.0e-3 $[x10^{-6}m]$: Radius
3 : Starts in the cytoplasm
3 : diffuses in the cytoplasm
0.1 $[(10^{-6}m)^2/s]$: Diffusion coefficient
0.0 $[(10^{-6}m)/s]$: velocity along cytoskeleton
Trans_3 : Name
0.0 0.0 0. : Color in in RGB

*** (22) Trans_4 (free in cytosol) ***
0 : Initial Number
5.0e-3 $[x10^{-6}m]$: Radius
3 : Starts in the cytoplasm
3 : diffuses in the cytoplasm
0.1 $[(10^{-6}m)^2/s]$: Diffusion coefficient
0.0 $[(10^{-6}m)/s]$: velocity along cytoskeleton
Trans_4 : Name
0.0 0.0 0. : Color in in RGB

*** (23) Trans_5 (free in cytosol) ***
0 : Initial Number
5.0e-3 $[x10^{-6}m]$: Radius
3 : Starts in the cytoplasm
3 : diffuses in the cytoplasm
0.1 $[(10^{-6}m)^2/s]$: Diffusion coefficient
0.0 $[(10^{-6}m)/s]$: velocity along cytoskeleton
Trans_5 : Name
0.0 0.0 0. : Color in in RGB

*** (24) Trans_6 (free in cytosol) ***
0 : Initial Number
5.0e-3 $[x10^{-6}m]$: Radius
3 : Starts in the cytoplasm
3 : diffuses in the cytoplasm
0.1 $[(10^{-6}m)^2/s]$: Diffusion coefficient
0.0 $[(10^{-6}m)/s]$: velocity along cytoskeleton
Trans_6 : Name
0.0 0.0 0. : Color in in RGB

*** (25) Motor catcher 1 ***
0 : Initial Number
5.0e-3 $[x10^{-6}m]$: Radius
1 : Starts in the plasma membrane

1 : diffuses in the plasma membrane
0.1 $[(10^{-6}m)^2/s]$: Diffusion coefficient
0.0 $[(10^{-6}m)/s]$: velocity along cytoskeleton
Tcatch_1 : Name
0.0 0.0 0. : Color in in RGB

*** (26) Motor catcher 2 ***
0 : Initial Number
5.0e-3 $[x10^{-6}m]$: Radius
1 : Starts in the plasma membrane
1 : diffuses in the plasma membrane
0.1 $[(10^{-6}m)^2/s]$: Diffusion coefficient
0.0 $[(10^{-6}m)/s]$: velocity along cytoskeleton
Tcatch_2 : Name
0.0 0.0 0. : Color in in RGB

*** (27) Motor catcher 3 ***
0 : Initial Number
5.0e-3 $[x10^{-6}m]$: Radius
1 : Starts in the plasma membrane
1 : diffuses in the plasma membrane
0.1 $[(10^{-6}m)^2/s]$: Diffusion coefficient
0.0 $[(10^{-6}m)/s]$: velocity along cytoskeleton
Tcatch_3 : Name
0.0 0.0 0. : Color in in RGB

*** (28) Motor catcher 4 ***
0 : Initial Number
5.0e-3 $[x10^{-6}m]$: Radius
1 : Starts in the plasma membrane
1 : diffuses in the plasma membrane
0.1 $[(10^{-6}m)^2/s]$: Diffusion coefficient
0.0 $[(10^{-6}m)/s]$: velocity along cytoskeleton
Tcatch_4 : Name
0.0 0.0 0. : Color in in RGB

*** (29) Motor catcher 5 ***
0 : Initial Number
5.0e-3 $[x10^{-6}m]$: Radius
1 : Starts in the plasma membrane
1 : diffuses in the plasma membrane
0.1 $[(10^{-6}m)^2/s]$: Diffusion coefficient
0.0 $[(10^{-6}m)/s]$: velocity along cytoskeleton
Tcatch_5 : Name
0.0 0.0 0. : Color in in RGB

*** (30) Motor catcher 6 ***
0 : Initial Number
5.0e-3 $[x10^{-6}m]$: Radius
1 : Starts in the plasma membrane
1 : diffuses in the plasma membrane
0.1 $[(10^{-6}m)^2/s]$: Diffusion coefficient
0.0 $[(10^{-6}m)/s]$: velocity along cytoskeleton
Tcatch_6 : Name
0.0 0.0 0. : Color in in RGB

*** (31) Coat catcher 1 ***
0 : Initial Number
5.0e-3 $[x10^{-6}m]$: Radius
1 : Starts in the plasma membrane
1 : diffuses in the plasma membrane
0.1 $[(10^{-6}m)^2/s]$: Diffusion coefficient
0.0 $[(10^{-6}m)/s]$: velocity along cytoskeleton
Ccatch_1 : Name
0.0 0.0 0. : Color in in RGB

*** (32) Coat catcher 2 ***
0 : Initial Number
5.0e-3 $[x10^{-6}m]$: Radius
1 : Starts in the plasma membrane
1 : diffuses in the plasma membrane
0.1 $[(10^{-6}m)^2/s]$: Diffusion coefficient
0.0 $[(10^{-6}m)/s]$: velocity along cytoskeleton
Ccatch_2 : Name
0.0 0.0 0. : Color in in RGB

*** (33) Cargo 1 ***
0 : Initial Number
5.0e-3 $[x10^{-6}m]$: Radius
1 : Starts in the plasma membrane
1 : diffuses in the plasma membrane
0.01 $[(10^{-6}m)^2/s]$: Diffusion coefficient
0.0 $[(10^{-6}m)/s]$: velocity along cytoskeleton
*Ste2p*a* : Name
1.0 0.0 0. : Color in in RGB (red)

*** (34) Cargo 2 ***
0 : (16000 for endocytosis 2-compartment test, 10000 for combined vesicle and signaling model)
5.0e-3 $[x10^{-6}m]$: Radius
1 : Starts in the plasma membrane
1 : diffuses in the plasma membrane
0.01 $[(10^{-6}m)^2/s]$: Diffusion coefficient
0.0 $[(10^{-6}m)/s]$: velocity along cytoskeleton
Ste2p : Name
0.0 0.0 0. : Color in in RGB

*** (35) Cargo 3 ***
0 : Initial Number
5.0e-3 $[x10^{-6}m]$: Radius
1 : Starts in the plasma membrane
1 : diffuses in the plasma membrane
0.1 $[(10^{-6}m)^2/s]$: Diffusion coefficient
0.0 $[(10^{-6}m)/s]$: velocity along cytoskeleton
Cargo_3 : Name
0.0 0.0 0. : Color in in RGB

*** (36) Cargo 4 ***
0 : Initial Number
5.0e-3 $[x10^{-6}m]$: Radius
1 : Starts in the plasma membrane
1 : diffuses in the plasma membrane
0.1 $[(10^{-6}m)^2/s]$: Diffusion coefficient

```
0.0 [(10^-6m)/s]       : velocity along cytoskeleton
Cargo_4                : Name
0.0 0.0 0.             : Color in in RGB
   *** (37) Cargo 5 ***
0                      : Initial Number
5.0e-3 [x10^-6m]       : Radius
1                      : Starts in the plasma membrane
1                      : diffuses in the plasma membrane
0.1 [(10^-6m)²/s] : Diffusion coefficient
0.0 [(10^-6m)/s]       : velocity along cytoskeleton
Cargo_5                : Name
0.0 0.0 0.             : Color in in RGB
   *** (38) Cargo 6 ***
0                      : Initial Number
5.0e-3 [x10^-6m]       : Radius
1                      : Starts in the plasma membrane
1                      : diffuses in the plasma membrane
0.1 [(10^-6m)²/s] : Diffusion coefficient
0.0 [(10^-6m)/s]       : velocity along cytoskeleton
Cargo_6                : Name
0.0 0.0 0.             : Color in in RGB
   *** (39) Signaling 1 ***
0                      : (20000 for the combined vesicle and signaling
model)
5.0e-3 [x10^-6m]       : Radius
3                      : Starts in the cytoplasm
3                      : diffuses in the cytoplasm
0.125 [(10^-6m)²/s]    : Diffusion coefficient
0.0 [(10^-6m)/s]       : velocity along cytoskeleton
MAPK_                  : Name
0.0 0.0 0.             : Color in in RGB
   *** (40) Signaling 2 ***
0                      : (6667 for the combined vesicle and signaling
model)
5.0e-3 [x10^-6m]       : Radius
2                      : Starts in the nucleus
2                      : diffuses in the nucleus
0.125 [(10^-6m)²/s]    : Diffusion coefficient
0.0 [(10^-6m)/s]       : velocity along cytoskeleton
MAPK_nucl.             : Name
0.0 0.0 0.             : Color in in RGB
   *** (41) Signaling 3 ***
0                      : Initial Number
5.0e-3 [x10^-6m]       : Radius
3                      : Starts in the cytoplasm
3                      : diffuses in the cytoplasm
0.125 [(10^-6m)²/s]    : Diffusion coefficient
1.0 [(10^-6m)/s]       : velocity along cytoskeleton (towards the
center, only if binding is specified)
MAPKp                  : Name
0.0 0.5 0.             : Color in in RGB (dark green)
   *** (42) Signaling 4 ***
0                      : Initial Number
5.0e-3 [x10^-6m]       : Radius
2                      : Starts in the nucleus
2                      : diffuses in the nucleus
0.125 [(10^-6m)²/s]    : Diffusion coefficient
0.0 [(10^-6m)/s]       : velocity along cytoskeleton
MAPKp_nucl.   : Name
0.0 0.0 0.             : Color in in RGB
```

Nuclear Import/Binding to Plasmamembran/Cytoskeleton

```
***definition: structure type, molecule type, rate***
0                      : Number of binding / Shuttling reactions (2 / 4
for the endocytosis / and signaling model)
1  19  13  0.015 [(10^-6m)/s]: Binding to the plasma membrane(1) of
(19=trans1_free) ==> (13=trans1_bound) with the specified rate
constant
1  3   5   0.36 [(10^-6m)/s]: Binding to the plasma membrane(1) of
(3=coat1_free) ==> (5=coat1_bound) with the specified rate constant
2  39  40  0.16 [(10^-6m)/s]: Binding to the nucleus(2) of (39=MAPK)
==> (40=MAPK_nucl.) with the specified rate constant
2  41  42  0.16 [(10^-6m)/s]: Binding to the nucleus(2) of (41=MAPKp)
==> (42=MAPKp_nucl.) with the specified rate constant
5.0 0.0                : general motor binding constant [(10^-6m)²/s] and
unbinding constant [1/s]
```

Stop Conditions

```
5000                   : Number of particles of species
1                      : that have to be überschritten (always false,
never stop)
```

Reactions:

```
F false                : there are no reactions
```

Clustering:

```
F false                : there is no clustering
```

For the endocytosis 2-compartment test and for the combined vesicle and signaling model the reaction part has to be replaced by:

```
T true                : there are reactions
Monomolecular / First-Order Reactions:
7                     : Number of first order reactions:
34 (Ste2p)            : Activation
1 33 (Ste2p*a)        :
0.025 [1/s]           : (0.25 [1/s] for the endocytosis 2-compartment test) Activation rate constant
13 (Trans1)           : Unbinding from plasma membrane
1 19 (Trans1 in cytoplasm):
0.01   [1/s]          : Rate constant
14 (Trans2)           : Unbinding from plasma membrane
1 20 (Trans2 in cytoplasm):
0.01   [1/s]          : Rate constant
5 (Coat1)             : Unbinding from plasma membrane
1 3 (Coat1 in cytoplasm):
0.25   [1/s]          : Rate constant
41 (MAPKp)            : Dephosphorylation in the cytoplasm
1 39 (MAPK)           :
0.2 [1/s]             : Rate constant
42 (MAPKp)            : Dephosphorylation in the nucleus
1 40 (MAPK)           :
0.05 [1/s]            : Rate constant
40 (MAPK_nucl.)       : Nucleo-Cytoplasmic Shuttling
1 39 (MAPK)           :
0.018 [1/s]           : Rate constant
42 (MAPKp_nucl.)      : Nucleo-Cytoplasmic Shuttling
1 41 (MAPKp)          :
0.013 [1/s]           : Rate constant
Bimolecular / Second-Order Reactions:
1                     : Number of second order reactions:
33 39                 : (Ste2p*a) + (MAPK)
2                     : ==> (Number of products)
33 41                 : (Ste2p*a) + (MAPKp)
2.0e6 0.0 0.0         : Rate constant [1/M 1/s], MM-Kinetics (no), Gradient in x (no)
```

Vesikel-Setup.txt:

```
General Properties:
0.25   [(10^-6m)/s]   : velocity of the actin boost
0.3    [(10^-6m)/s]   : local search velocity (depreciated, but still in this file)
0.5    [(10^-6m)/s]   : velocity of vesicle transport with motor proteins (is affected by the tug of war)
0.2     [(10^-6m)]    : threshold of the local search velocity (depreciated)
0.5 [1/s]             : Coat_depolymerization_rate constant (4.0 [1/s] for the "dipole" and directly connected model)
4                     : motor protein reference number for tug of war
25.e-3 [10^-6m ]      : standard vesicle radius = 25nm
4.e5 [1/M 1/s]        : kFusion0 (per effective snare pair)
150e-3 [s]            : TFusion0 (per effective snare pair)
5.e-3 [10^-6m ]       : d_max (cutoff-distance for the SNARE-Interacton)
Numbers
6   1000              : No. of vesicle types and max No. of vesicles
2 4 6 14              : No. of coat-, snare-, motor-, and cargo-types
Vesicle Machinery Interaction Rate constants:
 (1)     (2)     (3)     (4) ...
3.33     3.33 : Coat-Coat interaction for coat 1 and coat 2
0.001    0.0001    0.001    : global rate modifier for all coat-x interactions and two budding seed probabilities per timestep. The budding seed probabilities reduce the rate at which new buds are created in the vesicles (0.001   0.001   0.001 for the endocytosis simulations)
Coat-Snare interaction [(10^-6m)²/s], note the prefactor 0.001 from above:
0.70    0.00    0.07    0.00     (coat1-snare_i, i=1,4) (2.5 ... for the endocytosis simulations)
0.07    0.00    0.70    0.00     (coat2-snare_i, i=1,4) (2.5 ... for the endocytosis simulations)
Coat-Motor interaction [(10^-6m)²/s], note the prefactor 0.001 from above:
7.00    7.00    0.00    0.00     0.00    0.00    (coat1-motor_i, i=1,6)
7.00    7.00    0.00    0.00     0.00    0.00    (coat2-motor_i, i=1,6)
Coat-Cargo interaction (for membrane bound cargo) [(10^-6m)²/s], note the prefactor 0.001 from above:
1.5     0.1     0.0     0.0     0.0     0.0     0.05    0.5     0.0
0.0     0.0     0.0     0.3     0.2     (coat1-cargo_i, i=1,14)
0.1     1.5     0.0     0.0     0.0     0.0     0.5     0.05    0.0
0.0     0.0     0.0     0.2     0.3     (coat2-cargo_i, i=1,14)
Coat-Cargo interaction (for luminal cargo) [1/M 1/s], note the prefactor 0.001 from above:
0.0     0.0     0.0     0.0     0.0     0.0     0.0     0.0     0.0
0.0     0.0     0.0     0.0     0.0     (coat1-cargo_i, i=1,14)
```

0.0 0.0 0.0 0.0 0.0 0.0 0.0 0.0 0.0
0.0 0.0 0.0 0.0 0.0 (coat2-cargo_i, i=1,14)

Snare1 Snare2 Snare3 Snare4 :SNARE-SNARE-Interaction-Matrix

0.0 2.0 0.0 0.5 **Snare1**
2.0 0.0 0.5 0.0 **Snare2**
0.0 0.5 0.0 2.0 **Snare3**
0.5 0.0 2.0 0.0 **Snare4** (the endocytosis models used 2.0 instead of 0.5 on the diagonal)

Definition of Vesicle Types and Products

2 6 : number of definitions, default type if not defined
2 2 : fusions with 2 (e.g. ER or Endosome) inherit type 2
3 3 : fusions with 3 (e.g. Golgi) inherit type 3

Budding Products:

2 6 : number of definitions, default type if not defined
2 4 : vesicles from ER are type 4
3 5 : vesicles from Golgi are type 5

First order Reaction in Vesicles:

8 : first order reactions // Coat 1 unbinding
1 1 : indx 1, class 1 (=coat_1) :: unbinding reaction
1.0 [1/s] : rate constant
1 : no. of product molecules
3 : molecule indx 3
0 : of class 0 = individually tracked particle

Coat 2 unbinding reaction:
2 1 : indx 2, class 1 (=coat_2) :: unbinding reaction
1.0 [1/s] : rate constant
1 : no. of product molecules
4 : molecule indx 4
0 : of class 0 = individually tracked particle

Motor 1 unbinding reaction:
1 4 : indx 1, class 4 (=motor_1) :: unbinding reaction
0.01 [1/s] : rate constant
1 : no. of product molecules
19 : molecule indx 19
0 : of class 0 = individually tracked particle

Motor 2 unbinding reaction:
2 4 : indx 2, class 4 (=motor_2) :: unbinding reaction
0.01 [1/s] : rate constant
1 : no. of product molecules
20 : molecule indx 20
0 : of class 0 = individually tracked particle

Cargo 1 deactivation reaction:
1 5 :
0.1 [1/s] : rate constant
1 : no. of product molecules
3 : molecule indx 3
5 : of class 5 = cargo molecule

Cargo 2 deactivation reaction:
2 5 : indx 2, class 1 (=coat_2) :: unbinding reaction
0.1 [1/s] : rate constant
1 : no. of product molecules
4 : molecule indx 4
5 : of class 5 = cargo molecule

Cargo 1 creation reaction from source species:
5 5 :
0.1 [1/s] : rate constant
2 : no. of product molecules
5 1 : molecule indx 5 (keep source) and new (1)
5 5 : of class 5 = cargo molecule

Cargo 2 creation reaction:
2 6 : indx 2, class 1 (=coat_2) :: unbinding reaction
0.1 [1/s] : rate constant
2 : no. of product molecules
6 2 : molecule indx 6 and 2
5 5 : of class 5 = cargo molecule

Second order Reactions:

0 : none ...

For the combined endocytosis and signaling model one second order reaction was added while the four cargo creation/destruction reactions were deleted:

Second order Reactions:

1 : One second order reaction:
1 5 : indx 1, class 5 (membrane bound cargo) = Ste2p*a
4 6 : indx 4, class 6 (luminal protein) = "an Enzyme"
1e4 [1/M 1/s] : Rate constant
3 : three products:
2 4 3 : indx 1 (Ste2p), indx 4 ("enzyme") indx 3 ("alpha",dissolved mating factor)
5 6 6 : class 5 (membrane bound), class 6 (luminal)
That's it

```
Vesikel-Partikel-Interface.txt
Lookup table from vesicle to particle framework
2              : Number of Coat species
3 5            : free coat species for coat1 and coat2
4 7            : bound coat species for coat1 and coat2
4              : Number of Snare species
9              : particle-species id for snare1
10             : particle-species id for snare2
11             : particle-species id for snare3
12             : particle-species id for snare4
6              : Number of Motor species
13             : particle-species id for motor1
14             : particle-species id for motor2
15             : particle-species id for motor3
16             : particle-species id for motor4
17             : particle-species id for motor5
18             : particle-species id for motor6
14             : No of Cargo molecules
33             : particle-species id for cargo1 (Ste2p*a)
34             : particle-species id for cargo2 (Ste2p)
35             : particle-species id for cargo3 (alpha factor)
36             : particle-species id for cargo4 (“enzyme”)
37             : particle-species id for cargo5
38             : particle-species id for cargo6
25             : particle-species id for cargo 7 = (trans-catcher1)
26             : particle-species id for cargo 8 = (trans-catcher2)
27             : particle-species id for cargo 9 = (trans-catcher3)
28             : particle-species id for cargo 10 = (trans-catcher4)
29             : particle-species id for cargo 11 = (trans-catcher5)
30             : particle-species id for cargo 12 = (trans-catcher6)
31             : particle-species id for cargo 13 = (coat-catcher1)
32             : particle-species id for cargo 14 = (coat-catcher2)
Vesicle-Particle Reactions [Input: particle species id for first
reactant, species id of class5 (membrane bound in vesicle) for
second reactant]
4              : No. of V-P-Reactions
3   13         : type3 = coat1 + 5.13 = coat_catcher1
20e5 [1/M/s]: rate constant
2              : No. of products
1   13         : molecule species id of product(s)
1   5          : molecule class(es): coat and cargo5.
Coat2_Catching:
4   14         : type4 = coat2 + 5.14 = coat_catcher2
20e5 [1/M/s]: rate constant
2              : No. of products
2   14         : molecule species id of product(s)
1   5          : molecule class(es): coat and cargo5.
Motor1_Catching:
19   7         : type19 = motor1 + 5.7 = motor_catcher1
20e4 [1/M/s]: rate constant
2           : No. of products
1   7          : molecule species id of product(s)
4   5          : molecule class(es): motor and cargo5.
Motor2_Catching:
20   8          : type20 = motor2 + 5.8 = motor_catcher2
20e4 [1/M/s]: rate constant
2              : No. of products
2   8          : molecule species id of product(s)
4   5          : molecule class(es): motor and cargo5.
Coat-Membrane Binding (depreciated, replaced by the coat-catcher
concept)
(1)    (2)     affinity of the Coat Types for the compartments
0.0    0.0   : affinity to compartment 1
0.0    0.0   : affinity to compartment 2
0.0    0.0   : affinity to compartment 3
0.0    0.0   : affinity to compartment 4
0.0    0.0   : affinity to compartment 5
0.0    0.0   : affinity to compartment 6
```

The endocytosis models need only coat2 and trans2 catching because coat1 and trans1 are directed to the plasma membrane where the catching process is defined in the particle framework.
For the signaling endosome the following additional signal activation reaction is specified:

```
Signal activation at the compartment:
39   1         : type39 = MAPK + 5.1 = Ste2p*a
1e6 [1/M/s]   : rate constant
2             : No. of products
41   1         : molecule species id of product(s)
0    5         : molecule class(es): still particle based and still
cargo5.
```

Initial Compartments for the 2-Compartment Models:

Vesicles_Initial_Compartments.txt

```
2              : Number of initial compartments
== (ER) ==     : Name of the compartment
2              : Type of the compartment in this simulation
1000           : Size (times the standard vesicle size)
-0.2 1.25 0.0 [10^-6 m] :  x y z distance to the center of the cell
0.0  0.9  0.0               : Color in RGB (here: green)
250   125    :  Number of Coat Molecules in the compartment (1..2)
48000 4800  4800  48000 : Number of SNARE molecules (1..4)
4000  2000  0     0     0     0   : Number of motors (1..6) (0 for diffusion only)
16000 0     0     0     16000 0   1333  667  0  0  0  0  1000  500
: Number of membrane bound cargo (1..14)
0 0 0 0 0 0 0 0 0 0 0 0 0 0   : Number of luminal cargo (1..14)
==(Golgi)=     : Name of the compartment
3              : Type of the compartment in this simulation
1000           : Size (times the standard vesicle size)
-0.2 -1.25 0.0 [10^-6 m] :  x y z distance to the center of the cell
0.9  0.0  0.0               : Color in RGB (here: red)
125   250    :  Number of Coat Molecules in the compartment (1..2)
4800  48000 48000 4800  : Number of SNARE molecules (1..4)
2000  4000  0     0     0     0   : Number of motors (1..6) (0 for diffusion only)
0     16000 0     0     0     16000 667  1333  0 0 0 0  500 1000  :
Number of membrane bound cargo (1..14)
0 0 0 0 0 0 0 0 0 0 0 0 0 0   : Number of luminal cargo (1..14)
```
===Special Cytoskeleton Structures

0 0

===Actin Cables

=== Vesicle Sources in the cell

0

For the directly connected compartments the Special Structures have to be defined as follows:

===Special Cytoskeleton Structures:

```
1       0    : 1 Connection, 0 Actin Cables
Connecting
2       3    : (Er-Golgi)
0.0001  0.0001   0.  0.  : coat-anchor-interaction at (2)
0.0001  0.0001   0.  0.  : coat-anchor-interaction at (3)
1  1  0  0  0  0      : motor-special_cytoskeleton-interaction
(here motor 1 and 2 are assigned to walk on the connection.
```

===Actin Cables

Initial Compartments for the Endocytosis-Exocytosis Test:

Vesicles_Initial_Compartments.txt

```
1              : Number of initial compartments
== (Endosome) ==     : Name of the compartment
2              : Type of the compartment in this simulation
1000           : Size (times the standard vesicle size)
0.0   0.0   0.0 [10^-6 m] :  x y z distance to the center of the cell
0.0   0.9   0.0             : Color in RGB (here: green)
0     250     :  Number of Coat Molecules in the compartment (1..2)
10000 0     0     4800 : Number of SNARE molecules (1..4)
0     4000  0     0     0     0   : Number of motors (1..6) (0 for diffusion only)
0     10000 0     0     0     0   1333  667  1333  0 0 0 0  500 1000
: Number of membrane bound cargo (1..14)
0 0 0 394 0 0 0 0 0 0 0 0 0 0   : Number of luminal cargo (1..14)
```
===Special Cytoskeleton Structures

0 0

===Actin Cables

=== Vesicle Sources in the cell

0

Initial Compartments for the Combined Vesicle and Signal Transduction model:

Vesicles_Initial_Compartments.txt

```
1              : Number of initial compartments
== (Endosome) ==     : Name of the compartment
2              : Type of the compartment in this simulation
1000           : Size (times the standard vesicle size)
-0.75 0.0   0.0 [10^-6 m] :  x y z distance to the center of the cell
0.0   0.9   0.0             : Color in RGB (here: green)
0     250     :  Number of Coat Molecules in the compartment (1..2)
7000  0     0     4800 : Number of SNARE molecules (1..4)
0     4000  0     0     0     0   : Number of motors (1..6)
0     5000  0     0     0     0   1333  667  1333  0 0 0 0  500 1000
: Number of membrane bound cargo (1..14)
0 0 0 394 0 0 0 0 0 0 0 0 0 0   : Number of luminal cargo (1..14)
```
===Special Cytoskeleton Structures

0 0

===Actin Cables

=== Vesicle Sources in the cell

0
